# Tailored Fluorescent Metal–Organic Frameworks Hybrid Membrane Sensor Arrays: Simultaneous and Selective Quantification of Multiple Antibiotics

**DOI:** 10.1002/advs.202502452

**Published:** 2025-04-26

**Authors:** Tongtong Ma, Qiao Huang, Lei Yuan, Shugang Yan, Yalin Mo, Yibin Ying, Yingchun Fu, Jinming Pan

**Affiliations:** ^1^ College of Biosystems Engineering and Food Science Zhejiang Key Laboratory of Intelligent Sensing and Robotics for Agriculture Zhejiang University Hangzhou 310058 China

**Keywords:** antibiotics, hybrid membrane, metal–organic framework, sensor array, simultaneous detection

## Abstract

Sensor array offers significant potential for rapid, high‐throughput antibiotic detection. However, cross‐reactivity‐based sensor arrays often lack accuracy, despite comprehensive data analysis; while traditional high‐affinity‐based sensors based on antibodies/aptamers frequently suffer from complicated design and poor robustness. Here, a filterable paper‐based fluorescent metal–organic frameworks (MOFs) sensor array is developed for one‐to‐one recognition and quantification of multiple antibiotics. Three representative MOFs are designed to exceptional affinity and specificity for the target antibiotic. A filtration‐assisted detection enhances sensitivity, achieving parts‐per‐billion (ppb)‐level detection in mixed solutions. The proposed approach integrates recognition and signal generation, streamlined 10‐min process. The robustness of the MOFs also enables direct detection in raw samples containing organic solvents, which is not achievable by conventional methods. Notably, the sensor array can be easily incorporated into a smartphone‐based portable device, coupled with a user‐friendly image analysis applet for one‐step extraction and quantitative detection in chicken samples. Leveraging MOFs’ versatility, this method can be extended to simultaneously detect a broad range of antibiotics, offering the potential for universal, high‐throughput accurate detection of various chemical targets.

## Graphical abstract

A filterable paper‐based fluorescent metal–organic frameworks (MOFs) sensor array is developed for one‐to‐one recognition and simultaneous quantification of multiple antibiotics. Three representative MOFs are strategically designed to exhibit exceptional affinity and specificity for the target antibiotic. Moreover, the filtration‐assisted detection method significantly enhances the sensor array's sensitivity and integrates recognition and signal generation into a single, streamlined 10‐min process.

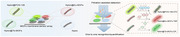

## 1 Introduction

Antibiotics, employed to treat human and animal diseases in medical, agriculture, and food industries, have generated serious antibiotic residues and caused considerable detrimental impacts on environmental and personal safety.^[^ ^1^, ^2^, ^3^, ^4^ ^]^ More seriously, the overuse of antibiotics will lead to superbug, increasing morbidity from bacterial infections and antibiotic resistance, with an estimated 10 million global deaths due to antibiotic infections per year by 2050.^[^ ^5^, ^6^ ^]^ By now, hundreds of antibiotics have been developed and broadly used, leading to multiple co‐residuals in most cases. For example, the Ministry of Agriculture of China claims 16 categories of antibiotics that should be monitored to ensure food safety.^[^ ^7^ ^]^ Hence, high‐throughput and accurate detection of antibiotics has garnered substantial attention. Typical laboratory assays, such as high‐performance liquid chromatography‐mass spectra (HPLC‐MS) offer precise and high‐throughput detection.^[^ ^8^ ^]^ However, they face significant challenges in achieving real‐time, in‐field detection due to poor portability and expensive instruments. Bio‐affinity‐based assay methods offer improvements in portability^[^ ^9^, ^10^ ^]^; however, they generally rely on tedious separation, recognition, and signal‐generation processes; the poor stability of biological antibodies or aptamers also leads to strict storage requirements and limit their application in impoverished and underdeveloped regions. Moreover, conventional biological assays are typically applied to the detection of a single type, with a low detection efficiency and throughput. Therefore, developing convenient and efficient methods that can not only detect different classes of antibiotics but also quantify their different forms is highly anticipated.

Given the complex mixture of the accumulated antibiotics, it is imperative to use array‐based sensors that exploit different interactions between identification materials and analytes with desirable specificity for selective detection. Recently, the sensor array has been regarded as one of the most promising multiple detection methods due to its the high‐throughput, rapid response, ease of operation, and portability.^[^ ^11^, ^12^, ^13^, ^14^ ^]^ Despite recent advances (Table S1, Supporting Information), the sensor arrays primarily rely on cross‐reactivity and generally require a large number of sensing elements, rather than providing accurate one‐to‐one direct signals.^[^ ^15^, ^16^ ^]^ Therefore, specificity in complex samples and accurate quantification are hardly achievable, though complementary data analysis models have been developed to improve their applicability.^[^ ^17^ ^]^ Clearly, the exploration of recognition elements with preferable specificity and affinity and the innovation of a new sensing model is of high urgency.

Biological recognition elements, such as antibodies, enzymes, and nucleic acids/aptamers, are widely used due to their high affinity and specificity; however, their short shelf life, high cost, and low stability have caused solid hindrances in many applications. Moreover, complicated signal generation and amplification processes are generally required due to the lack of signal‐related properties of biological elements. Alternatively, chemical antibodies have drawn increasing attention due to their similar affinity and specificity as biological antibodies but significantly improved stability and cost efficiency, as well as diversified functions. MOFs offer efficient recognition sites through programable 3D configurations, exhibit superior porosity, stability, function ability, and tolerance to a wide range of pH and organic solvents, and have been regarded as one of the most promising alternatives to biological recognition elements.^[^ ^18^, ^19^, ^20^ ^]^ Especially, MOFs with responsibility powered by fluorescence and electric properties have evolved to be novel recognition and signal generation all‐in‐one elements for high integration sensing.^[^ ^21^, ^22^, ^23^ ^]^ Fluorescent MOFs powders in solution have been explored to develop sensors with superior sensitivity and simplicity.^[^ ^24^, ^25^ ^]^ However, the usage of powers also significantly lowered facility and reproducibility due to the poor dispersibility of powders in solution (suspension), more importantly, limited the potential for multiple detection of different targets.^[^ ^26^ ^]^ The exploitation of fluorescent MOFs to solid substrates to develop a sensor array chip should open a new way to well address the above shortcomings and enable a new strategy for facile and multiple detection.

**Figure 1:**
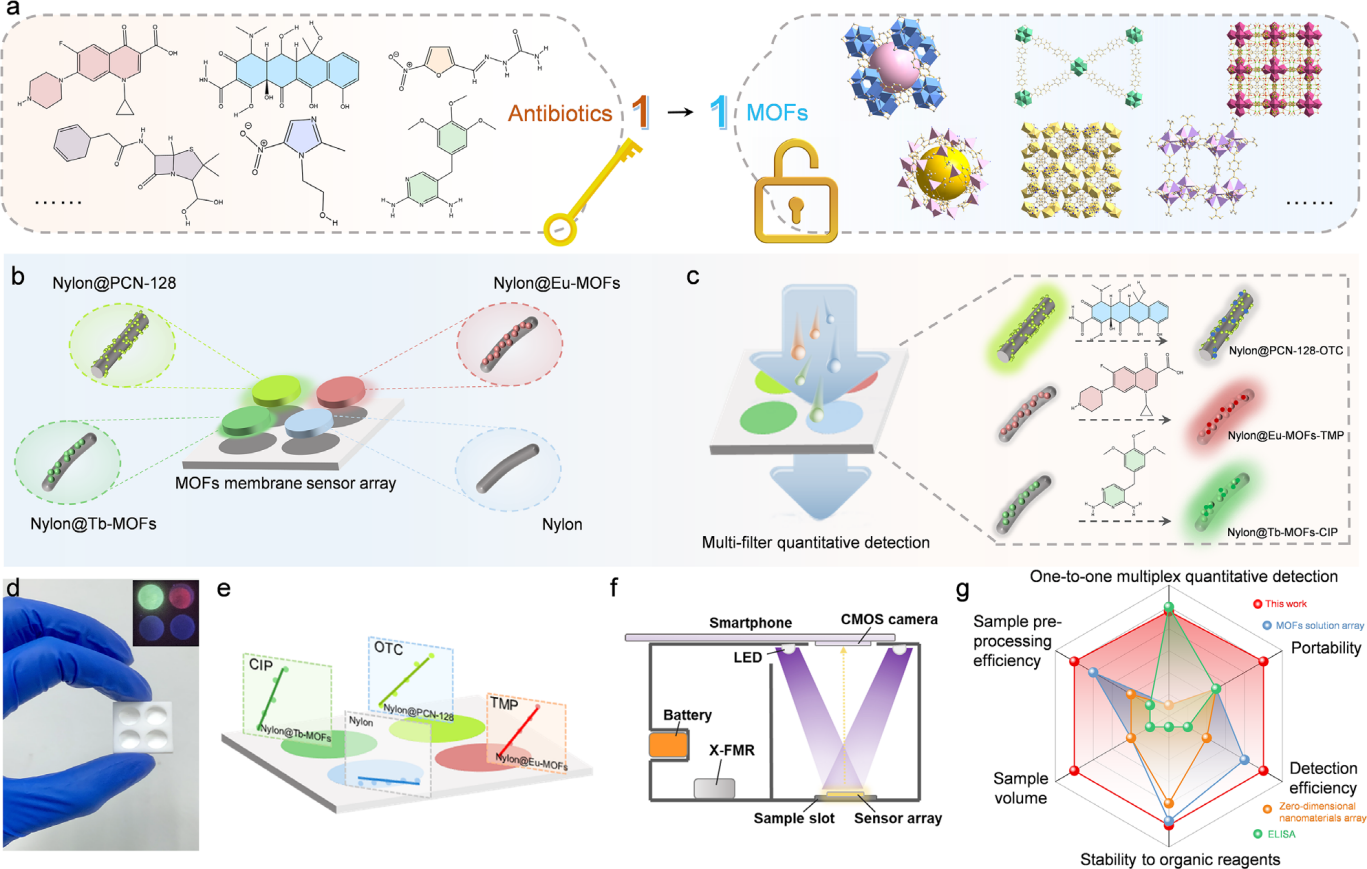
One‐to‐one quantitative detection of multiple antibiotics based on MOFs membrane sensor array. a) Illustration of one‐to‐one recognition and response between MOFs and target antibiotics. b) A schematic representation of the MOFs membrane sensor array, comprising Nylon@PCN‐128, Nylon@Eu‐MOFs, Nylon@Tb‐MOFs, and nylon, and c) its application for filtration‐based one‐to‐one quantitative detection of multiple antibiotics. d) Physical and fluorescence images of the MOFs membrane sensor array. e) Illustration of quantitative detection based on the different areas. f) Light path diagram of homemade portable device. g) Performance comparison between the MOFs membrane sensor array and other common sensor arrays.

Herein, we proposed a paper‐based hybrid membrane sensor array by integrating multiple fluorescent MOFs on a filter membrane substrate to develop a one‐to‐one recognition and direct quantification method for the simultaneous detection of multiple antibiotics. By leveraging the distinct chemical structures of antibiotics and the advantages of fluorescent MOFs with large specific surface areas and visual response capabilities, we designed three types of MOFs (PCN‐128(Zr), Eu‐MOFs, and Tb‐MOFs) to enable superior affinity and specificity to oxytetracycline (OTC), trimethoprim (TMP), and fluoroquinolones, respectively (**Figure** **1**a). The hybrid membrane was fabricated by modifying MOFs on the filter membrane using a rational combination of microwave‐assisted solvothermal synthesis and vacuum‐assisted filtration methods. Then, the facile detection of three types of antibiotics was enabled by simply filtrating sample solution through the membrane. The fluorescence intensity (FI) change of each MOF module was recorded before and after the filtration for quantification by using a smartphone‐based portable image‐capturing system (Figure 1b–e). Importantly, by integrating the high absorbability, specificity, and responsive ability of the MOFs with the inherent porosity of the filter membrane, a filtration‐enabled detection method was elaborated, namely, the recognition and signal generation were simultaneously realized during the filtration. The method eliminated tedious independent recognition, separation, labeling, and signal generation processes, making the entire detection into one procedure that can be completed within 10 min. Moreover, conventional ELISA methods generally take a limited volume of sample less than 200 µL, which significantly restricts the amount of target for detection; while the new filtration model extended the sample volume to more than 5 mL, which obviously increased the amount of target to enhance detection performance. Due to the high tolerance of MOFs to organic solvents, the hybrid membrane enabled the direct detection in a raw sample solution containing high‐concentration extraction organic solvents, which also eliminated tedious sample treatment procedures such as repeated extraction and nitrogen blowing, benefiting an operation‐, time‐ and solvent‐saving way. Furthermore, the homemade smartphone‐based portable monitoring system was designed to be compatible with the membrane sensor array, which was successfully applied for the rapid simultaneous detection of three antibiotics with the limitation of detection of ppb level in chicken samples (Figure 1f). Compared to conventional sensor arrays, this MOFs membrane sensor array showed a series of advantages of high specificity, portability, sample treatment and detection efficiency, stability, etc., making it a superior method over analogs (Figure 1g).

## 2 Results and Discussion

### 2.1 Characterizations and Sensing Performance of MOFs

**Figure 2:**
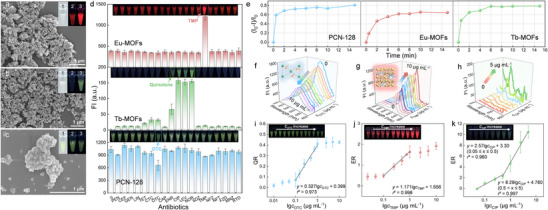
Characterizations and sensing performance of different fluorescent MOFs. SEM images of a) Eu‐MOFs, b) Tb‐MOFs, and c) PCN‐128, insets are the photographs of these MOFs suspensions under daylight (1), the fluorescence changes before (2) and after (3) the response to target antibiotics. d) Fluorescent responses of three MOFs to different antibiotics. e) Adsorption quantity of different MOFs at different times (0–15 min). Fluorescent emission spectra of f) PCN‐128, g) Eu‐MOFs, and h) Tb‐MOFs to different concentrations of target antibiotics. The corresponding calibration plots of i) PCN‐128, j) Eu‐MOFs, and k) Tb‐MOFs. Insets are the corresponding photographs of different MOFs under UV light for visualization.

Highly specific and sensitive recognition‐response materials are key to achieving exceptional one‐to‐one multiple quantitative detection. To achieve specific and sensitive rapid detection, we synthesized three representative fluorescent MOFs (PCN‐128, Eu‐MOFs, and Tb‐MOFs) according to the structures of target antibiotics. Scanning electron microscope (SEM) (**Figure** **2**a–c), Fourier transform infrared spectroscopy (FT–IR) (Figures S4 and S5, Supporting Information), thermogravimetric analysis (TGA) (Figure S6, Supporting Information), and N_2_ adsorption–desorption isotherms (Figure S7, Supporting Information) were used to characterize the successful preparation. Eu‐MOFs, Tb‐MOFs, and PCN‐128 exhibited particle morphologies with nanometer or micrometer sizes. Three MOFs could be easily dispersed in water under sonication to form suspensions (insets of Figure 2a–c), which presented bright fluorescence under the excitation of ultraviolet (UV) light and underwent significant fluorescence change in the presence of target antibiotics. Optimization of synthesis conditions (Figures S1 and S2, Supporting Information), fluorescence spectra (Figure S3, Supporting Information), FT–IR, TGA, and N_2_ adsorption–desorption isotherms also showed clear proof of successful preparation, as detailed in the Supporting Information.

To highlight the high specificity of the three representative synthesized MOFs, we tested the responses of MOFs to 19 types of conventional antibiotics including penicillin (PEN), cefixime (CEF), gentamicin (GEN), lincomycin (LIN), tetracycline (TET), chlortetracycline (CTC), OTC, chloramphenicol (CAP), enrofloxacin (ENR), ciprofloxacin (CIP), levofloxacin (LEV), norfloxacin (NOR), sulfadiazine (SDZ), TMP, nitrofurazone (NFZ), nitrofurantoin (NFT), furazolidone (FZD), metronidazole (MNZ), and rimantadine (RTD). Note that the specificity is generally tested against around six types of interfered antibiotics in references, which may be not able to provide solid evidence because of the high diversity of antibiotics.^[^ ^27^, ^28^, ^29^ ^]^ In this study, the major types of antibiotics have been covered to ensure solid specificity. Besides, we tested five common amino acids (lysine (Lys), glutamic acid (E), aspartic acid (Asp), glycine (Gly), and proline (Pro)) along with three sugars (glucose (Glu), galactose (Gal), and fructose (Fru)) and two heavy metal ions (Cd^2+^ and Pb^2+^). As shown in Figure 2d and Figures S8 and S9 (Supporting Information), among the 19 kinds of antibiotics and 10 types of non‐antibiotics, only TMP and fluoroquinolone antibiotics such as CIP markedly increased the FI of Eu‐MOFs and Tb‐MOFs, respectively; in contrast, OTC significantly decreased the FI of PCN‐128, which established a robust foundation for the excellent specificity of multiplexed simultaneous detection. The high specificity of the synthesized MOFs could be primarily attributed to their strong adsorption capacity and the energy/electron transfer effects with the target antibiotics. Specifically, the hydroxyl‐rich OTC formed strong hydrogen bonds with the hydroxyl groups of PCN‐128, leading to tight binding (Figure S10, Supporting Information). Fluorescence lifetime measurements, spectral overlap between PCN‐128 and OTC, and density functional theory (DFT) calculations suggest that the fluorescence quenching phenomenon is primarily caused by the inner filter effect and photoinduced electron transfer mechanism between PCN‐128 and OTC.^[^ ^30^, ^31^ ^]^ Additionally, TMP and CIP acted as antenna molecules, respectively, transferring energy to Eu^3+^, and Tb^3+^ and thereby inducing fluorescence enhancement.^[^ ^32^, ^33^ ^]^ Moreover, the detailed mechanistic investigation of the three MOFs is shown in Figures S11–S13 (Supporting Information). Therefore, the three representative synthesized MOFs hold great potential as excellent materials for one‐to‐one identification and direct quantitative detection.

The adsorption amount and kinetics of different antibiotics are the key prerequisites for rapid quantitative detection. Figure 2e and Figure S14 (Supporting Information) showed the adsorption amount of antibiotics (initial concentration: 10 µg mL^−1^) rapidly increased along with the reaction time and became constant in 6 min, reaching an adsorption amount of 7.40 µg mL^−1^ (PCN‐128), 6.18 µg mL^−1^ (Eu‐MOFs), and 7.85 µg mL^−1^ (Tb‐MOFs), respectively, which is significantly faster than most MOFs.^[^ ^29^, ^34^, ^35^ ^]^ Moreover, different adsorption kinetics of three synthesized MOFs also provided solid support for the high specificity. Detailly, when the adsorption rate reached 60%, PCN‐128 required only 0.5 min, while Eu‐MOFs and Tb‐MOFs required ≈6 and 2 min, respectively. Here, the various adsorption efficiencies of the three MOFs are attributed to differences in the strength of interactions, such as electrostatic action and π–π interactions, between the framework structures and the antibiotics.

Based on the optimal concentration (detailed in Figure S15, Supporting Information) and detection time in 10 min (Figure S16, Supporting Information) of the three MOFs, we validated the fluorescence detection capability for the target antibiotics. As shown in Figure 2f–h, the FI of the MOFs solution gradually decreased or increased along with the increase of target antibiotics concentration. The quenching rate (QR) of PCN‐128 exhibited a good linear relationship to the logarithmic molar volume of the concentration of OTC (Figure 2i). A linear detection range (LDR) was observed between the concentrations from 0.1 to 1 µg mL^−1^, and the limit of detection (LOD) was calculated to be 0.06 µg mL^−1^ (*S*/*N* = 3). Inversely, the enhancement rates (ERs) of Eu‐MOFs and Tb‐MOFs enhanced as the concentration of TMP or CIP increased (Figure 2j,k). Similarly, the LODs for TMP and CIP reached 0.046 and 0.052 µg mL^−1^ according to the corresponding calibration plot, respectively (*S*/*N* = 3). Notably, the LODs of the three antibiotics were below the chicken products of national standards of China (GB31650‐2019, 0.2 µg mL^−1^ for OTC, 0.1 µg mL^−1^ for TMP and 0.1 µg mL^−1^ for CIP), demonstrating the considerable responsive ability of the three representatives MOFs toward corresponding antibiotics.

### 2.2 Preparation and Detection Performance of Fluorescent MOFs Membrane Sensors

**Figure 3:**
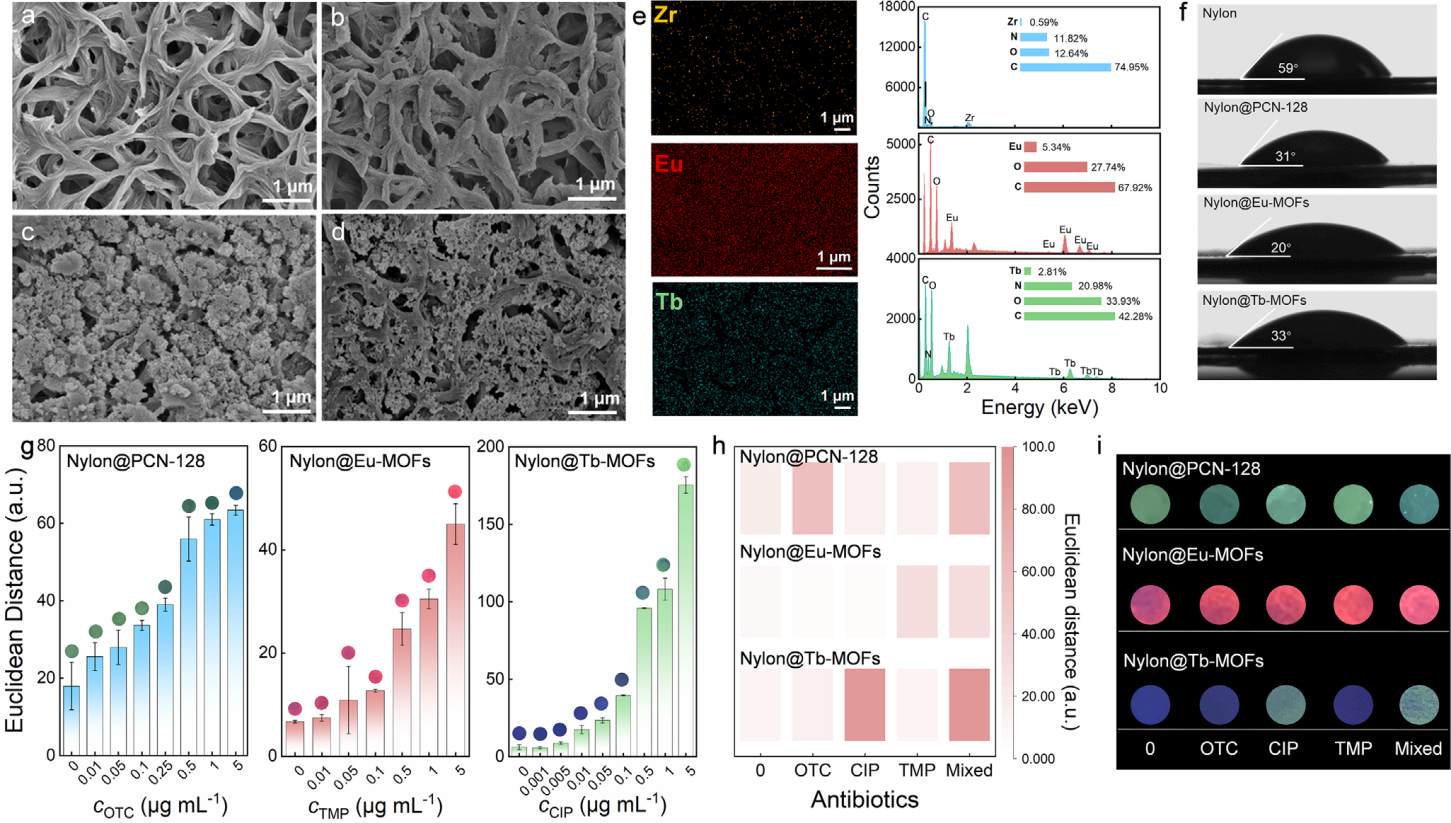
Characterizations and detection performance of fluorescent MOFs hybrid membrane sensors. SEM images of a) Nylon, b) Nylon@PCN‐128, c) Nylon@Eu‐MOFs, and d) Nylon@Tb‐MOFs. e) Elemental mapping and corresponding EDS data of different MOFs hybrid membranes. f) Water contact angle measurement of the membranes. g) ED values and physical images color difference of Nylon@PCN‐128, Nylon@Eu‐MOFs, and Nylon@Tb‐MOFs. h) ED heatmaps and i) physical images of the color difference of Nylon@PCN‐128, Nylon@Eu‐MOFs, and Nylon@Tb‐MOFs to different antibiotics at 1 µg mL^−1^.

Based on the three synthesized MOFs with high specificity, rapid adsorption behavior, and low detection limit, we prepared integrated hybrid membranes on commercial nylon fiber membranes to improve detection portability. The hybrid membrane was fabricated by modifying MOFs on the filter membrane using a rational combination of microwave‐assisted solvothermal synthesis and vacuum‐assisted filtration methods, which are detailed in Figure S17 (Supporting Information). SEM (**Figure** **3**a–d), FT–IR (Figure S18, Supporting Information), elemental mapping, and energy dispersive spectrometer (EDS) (Figure 3e) were used to characterize the successful preparation. Obviously, Eu‐MOFs and Tb‐MOFs particles covered the surface of the nylon membrane and effectively filled the micron pores of the membrane through hydrogen bonding interactions between the hydroxyl groups on nylon and the carboxyl/amino groups on the Eu‐MOFs/Tb‐MOFs (Figure 3c,d; Figure S18b,c, Supporting Information). Benefiting from the advantages of microwave‐assisted synthesis, Nylon@PCN‐128 was synthesized in just 10 min. Additionally, PCN‐128 grew along the fibers, enhancing the overall specific surface area of the membrane while preserving its filtration pores (Figure 3b). Moreover, elemental mapping and EDS results displayed the uniform distribution and the characteristic element of the Zr, Eu, and Tb in the hybrid membrane, with the amount of 0.59, 5.34, and 2.81 wt.%, respectively (Figure 3e). The water contact angle results further indicated a decrease in the contact angle on the top surface due to the presence of hydrophilic MOFs, which will amplify the interaction between the targets in the solution and MOFs hybrid membrane (Figure 3f). The above characterizations all confirmed the successful preparation of different MOFs hybrid membranes and laid a solid foundation for sensor array chips.

Upon the optimized microwave power of Nylon@PCN‐128 (Figure S19, Supporting Information) and the amount of Nylon@Eu‐MOFs/Nylon@Tb‐MOFs (Figure S20, Supporting Information), three MOFs hybrid membranes were examined in soaking mode to evaluate the detection performance. Notably, acetonitrile aqueous solution (ACN‐H_2_O, V(ACN): V(H_2_O) = 15:2) that is generally adopted as extraction reagent in the chicken sample was used as the reaction solution for the detection, replacing the laboratory's commonly used water or other mild buffer solutions. The FI changes of each hybrid membrane before and after the reaction were recorded using a smartphone‐based portable image‐capturing system and converted into Euclidean distance (ED) values for quantification. ED value is the straight‐line distance between two points in the red, green, and blue (RGB) color space. A larger ED value represents a stronger response to targets. As shown in Figure 3g, the ED values of the three hybrid membranes enlarged with the increasing concentrations of antibiotics, and the changes became visually detectable to the naked eye at concentrations exceeding 0.1 µg mL^−1^. Based on the calibration plots of the hybrid membranes, the LOD of the Nylon@PCN‐128, Nylon@Eu‐MOFs, and Nylon@Tb‐MOFs were calculated to be 0.0085, 0.0225 and 0.00136 µg mL^−1^, respectively, which were significantly lower than that based on MOFs powders (Figure S21, Supporting Information). Clearly, the excellent performance profited from the enrichment and the porous structure of the hybrid.

Based on the high specificity of the MOFs powders, the hybrid membranes are also expected to exhibit one‐to‐one selectivity for the target antibiotics. 1 µg mL^−1^ of OTC, TMP, CIP, and their mixed antibiotic solution were selected to react with different MOF membranes for selectivity verification. Figure 3h,i illustrated significant color changes in the MOFs hybrid membrane sensor images exclusively in the presence of the target antibiotic or the mixed solution. Furthermore, the precise ED values corresponding to these color changes are presented in Figure S22 (Supporting Information). The ED values before and after the detection were similar when exposed to the target antibiotic and mixed solution, and notably higher than those observed for the other two antibiotics. For instance, the ED values of Nylon@PCN‐128 before and after the reaction with OTC and the mixed solution were 58.705±1.798 and 56.678±7.652, respectively, while the ED values in ACN‐H_2_O, TMP, and CIP solution were 17.953±6.106, 15.268±2.506, and 14.202±6.121, respectively. It demonstrated that the MOFs on the hybrid membrane exhibited unique fluorescence changes only with specific target antibiotics. These properties underscore the one‐to‐one high specificity of the synthesized MOFs for antibiotic detection, which remained intact even after integration with the fiber membrane. This characteristic provided a robust foundation for achieving one‐to‐one multiplexed sensitive detection in sensor array chips.

### 2.3 Simultaneous Quantitative Detection of Fluorescent MOFs Membrane Sensor Array

**Figure 4:**
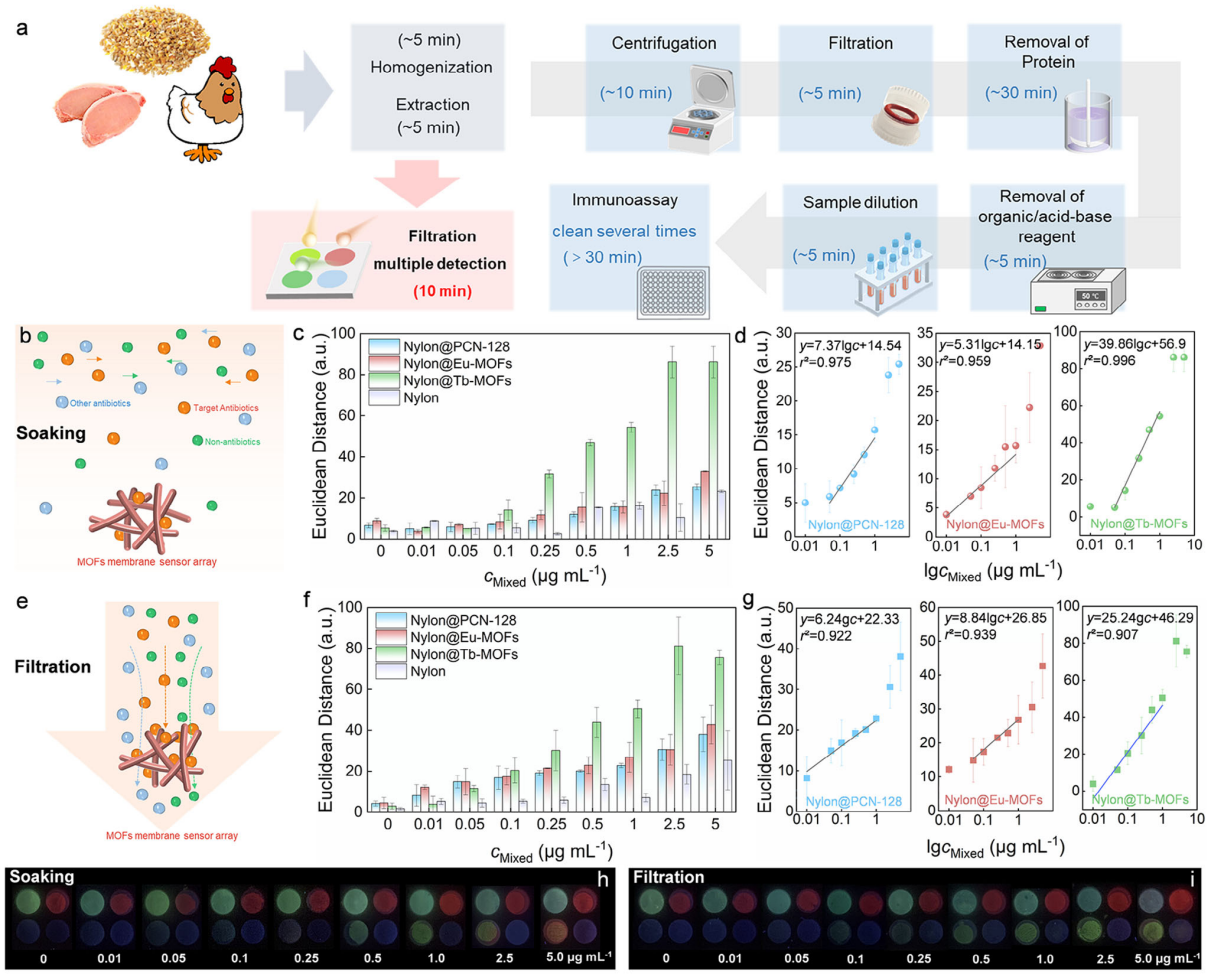
a) Illustration of the antibiotic extraction from solid samples and detection using the fluorescent MOFs membrane sensor array, in comparison with the immunoassay method. b) Scheme of the sensing process in soaking mode. c) Changes of ED values of MOFs membrane sensor array and d) the corresponding calibration plot of different regions to mixed antibiotics solutions in soaking mode. e) Scheme of the sensing process in filtration mode. f) Changes of ED values of MOFs membrane array sensor and g) the corresponding calibration plot of different regions to mixed antibiotics solutions in filtration mode. The corresponding photographs of MOFs membrane sensor arrays in soaking mode h) and filtration mode i) under UV light for visualization.

Owing to the high specificity and excellent fluorescence detection performance of the single MOFs hybrid membrane, different membranes are also highly demanded to be integrated, thereby synergistically optimizing properties for one‐to‐one direct multiple quantitative detection without the need for complex data model calculations. Although immunoassay methods have been developed for the simultaneous detection of multiple hazards,^[^ ^14^, ^36^ ^]^ the poor stability of biological recognition elements such as antibodies and the necessity for multi‐step sample preparation (more than 6 steps) impose significant challenges, further emphasizing the demand for simplified pretreatment and rapid, sensitive detection methods. MOFs membrane sensor array chips simultaneously realized the recognition and signal generation based on the fluorescent MOFs during the reaction, which eliminated tedious independent recognition, separation, labeling, and signal generation processes, making the entire detection into one procedure that can be completed within 10 min (**Figure** **4**a).

Subsequently, we utilized a custom‐designed support component (Figure S23, Supporting Information) to assemble the three MOFs hybrid membranes and a nylon membrane without MOFs into a handheld integrated membrane array chip. In this design, three MOFs membranes were individually employed for the recognition and fluorescence detection of target antibiotics in mixed solutions, while the bare nylon membrane served as a brightness adjustment control to minimize the influence of lighting during image acquisition with a smartphone. The response of the sensor array with high selectivity depended on the interaction between chemically responsive fluorescent MOFs and antibiotics as mentioned above. To evaluate the one‐to‐one multiplex detection performance of the MOFs membrane sensor array chip, a mixed solution of three antibiotics (OTC, TMP, and CIP) in ACN‐H_2_O at equal volumes was prepared to simulate real samples. Notably, the MOFs membrane sensor array chip required no additional processing and was directly soaked in the mixed antibiotic solution for 10 min. After UV excitation, the chip could be used for image acquisition. As shown in Figure 4b, during the soaking process, antibiotic molecules in the solution are in a disordered state, freely diffusing to the membrane where they are detected. Based on the high specificity of the MOFs chemical recognition elements toward target antibiotics and their unique fluorescence detection mechanism, the distinct MOFs‐responsive regions on the chip can accurately identify and precisely detect target antibiotics from mixed antibiotic solutions. Especially, similar to the single MOFs hybrid membrane, the ED values of different MOFs‐responsive regions increased with the concentration of the mixed solution, accompanied by more pronounced fluorescence color changes in the images (Figure 4c,h). The equal interaction probability of MOFs with different antibiotics in the mixed solution may lead to interference from highly responsive MOFs, which explains the red color observed in the Tb‐MOFs region at a concentration of 5 µg mL^−1^ in the mixed solution. Due to the one‐to‐one high selectivity of MOFs, it could be directly translated into a standard curve for the target antibiotic in mixed solutions, enabling quantitative analysis. As shown in Figure 4d, The LODs for OTC, TMP, and CIP were calculated to be 0.014, 0.023, and 0.042 µg mL^−1^ (*S*/*N* = 3), respectively, meeting the national standards of China (GB31650‐2019). Although the detection performance of the MOFs membrane sensor array chip is lower than that of a single MOFs membrane, a 10‐min reaction was sufficient for the preliminary screening of three antibiotics in a mixed solution, with no additional processing required, making the method simple and time‐efficient.

Furthermore, by integrating the high absorbability, specificity, and responsive ability of the MOFs with the inherent porosity of the filter membrane, a filtration‐enabled detection method was elaborated. A miniaturized vacuum filtration device compatible with the MOFs membrane sensor array was designed to simplify complex detection procedures, which enabled the simultaneous identification, adsorption, and response to three antibiotics in a mixed solution during a single filtration process (Figure S24, Supporting Information). Notably, molecules in the solution exhibited directional movement under the influence of filtration pressure, significantly suppressed the concentration gradient, and increased the concentration of antibiotics on the surface of the membrane (Figure 4e). Supported by filtration, other antibiotics, and impurities are filtered out while the target antibiotics are efficiently adsorbed by the MOFs on the membrane, inducing distinct fluorescence changes and enabling both efficient adsorption and rapid detection in a single step. Building on the directional movement of antibiotic molecules under filtration mode, the MOFs membrane sensor array chip demonstrated significantly improved detection performance for mixed solutions, particularly at low concentrations below 1 µg mL^−1^ (Figure 4f). This enhancement is primarily attributed to the increased contact opportunities between the MOFs on the membrane and the antibiotics, compared to the random molecular movement in soaking mode. Specifically, Nylon@PCN‐128, Nylon@Eu‐MOFs, and Nylon@Tb‐MOFs regions were linearly proportional to the concentration of mixed solution within the range of 0.01–1 µg mL^−1^, 0.05–1 µg mL^−1^, and 0.01–1 µg mL^−1^, respectively (Figure 4g). Moreover, based on the standard curves, the LODs for OTC, TMP, and CIP in the mixed solution were 0.001, 0.003, and 0.019 µg mL^−1^ (*S*/*N* = 3), respectively, all of which were more than two‐fold lower than those achieved in the soaking mode. Notably, the fluorescence images under filtration mode showed pronounced changes, with no mutual interference among the MOFs even at 5 µg mL^−1^, further underscoring the high specificity of each responsive region toward the target antibiotic and the absence of cross‐reactivity under filtration conditions (Figure 4i). Therefore, a MOFs membrane sensor array chip with high throughput, high sensitivity, and high efficiency was achieved in the filtration mode, showing a new mode of simultaneous quantitative detection.

### 2.4 Practical Application Capability of Fluorescent MOFs Membrane Sensor Array

**Figure 5:**
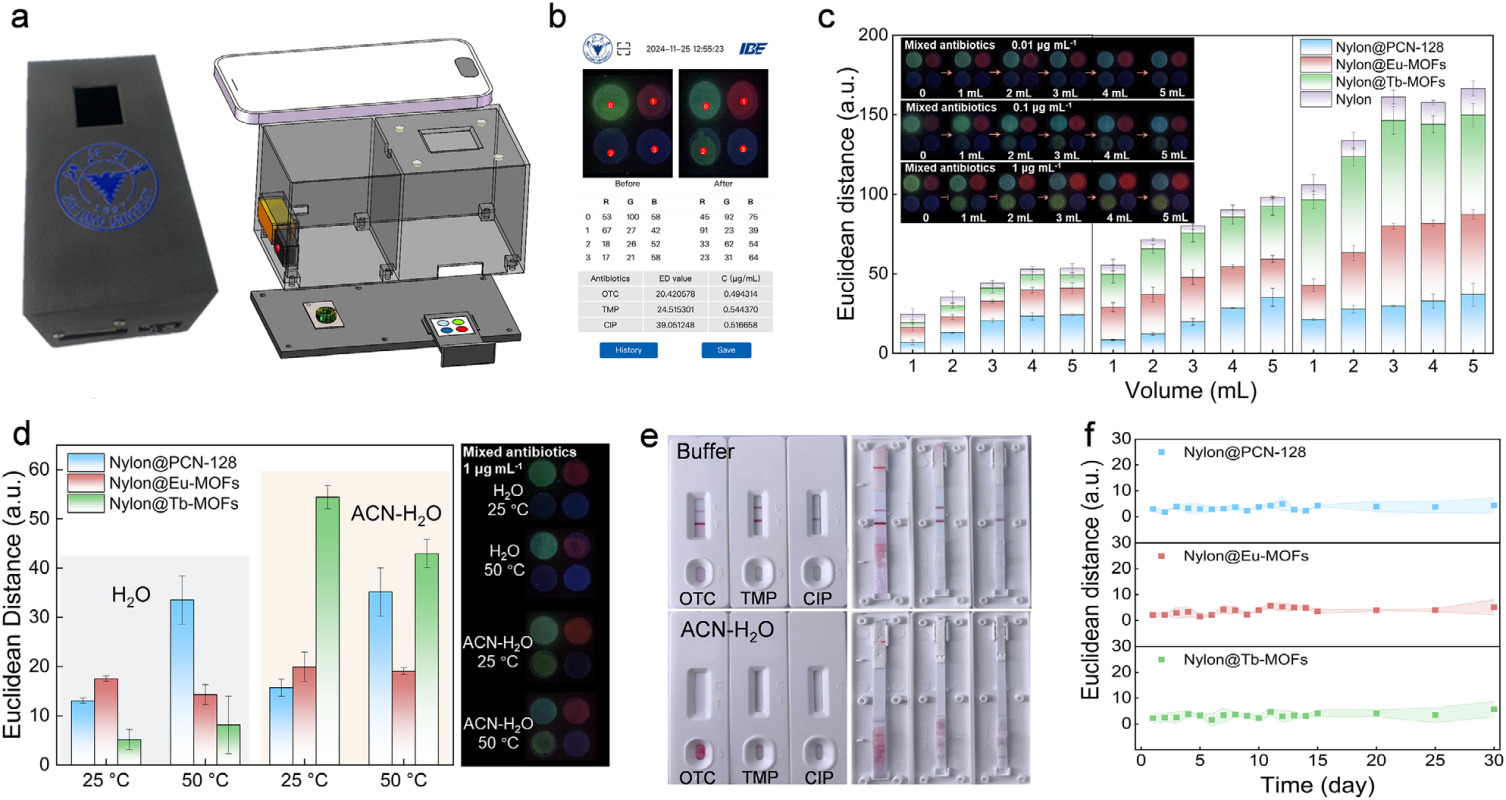
a) Image and composition of the smartphone‐based portable device. b) The home screen of custom‐developed image analysis applet. c) Changes of ED values to different volumes of mixed antibiotics solutions at 0.01, 0.1, and 1 µg mL^−1^. d) The tolerance of each response region of the MOFs membrane sensor array to ACN‐H_2_O and high‐temperature conditions. e) Sensing performance of immunochromatographic test strips for different antibiotics in buffer solution and ACN‐H_2_O. f) Storage stability. Insets are the corresponding photographs under UV light for visualization.

To achieve rapid on‐site detection, the MOFs membrane sensor array requires integration with portable signal acquisition devices and image analysis software, enabling efficient detection efficiency and portable signal analysis. We employed a homemade 3D‐printed device compatible with the array to form a portable analytical device for fluorescence excitation and image acquisition. **Figure** **5**a illustrates the basic components and light path diagram of the smartphone‐based 3D‐printed device, where the sensor array was inserted into the holder and excited by a built‐in LED. Subsequently, images captured using our system are processed by the custom‐developed image analysis applet, which extracts RGB values, calculates the ED values of the sensor array in the background, and directly outputted the concentration of each antibiotic, offering a convenient, rapid, and accurate solution without the need for manual data processing (Figure 5b). Most importantly, by integrating MOFs membrane sensor array with a smartphone platform, we realized high throughput and fast sensitive simultaneous detection of mixed antibiotics solution for the first time without biological recognition elements or model analysis. Furthermore, the filtration flux of the MOFs membrane sensor array was carried out by continuous filter mixed solution at concentrations of 0.01, 0.1, and 1 µg mL^−1^. As shown in Figure 5c, with increasing filtration flux (≤ 5 mL), the ED values for different regions of the MOFs membrane sensor array generally increased. Compared to traditional immunoassay methods, such as ELISA or immunochromatographic test strips, which typically restrict sample volumes to less than 200 µL, the novel filtration model based on MOFs membranes sensor array chip expanded the sample volume to over 5 mL, significantly increasing the target analyte quantity and improving detection performance.

Indeed, during the sample pretreatment process, complex conditions (e.g., organic solvents, high temperature) are common, coming from the pretreatment procedures such as sample extraction and the surrounding environment. These might disturb the normal operation of the sensor, particularly by affecting the receptors, thus creating a high demand for durable and robust receptors and sensors. Here, we chose ACN‐H_2_O solution (1 µg mL^−1^) and 50 °C as the organic reagent and temperature interference, respectively, to investigate the practical application capabilities of MOFs membrane sensor arrays.^[^ ^21^, ^37^ ^]^ As shown in Figure 5d, the stabilities of MOFs membrane array sensor under these conditions were validated with acceptable ED values, consistent with the fluorescence physical images (inset of Figure 5d). These profit from the chemical and thermal stabilities of MOFs, as well as the structure stability of the membrane array. It is noteworthy that the relatively low fluorescence response of Nylon@Tb‐MOFs in H_2_O may be attributed to the competitive coordination of water molecules with CIP.^[^ ^32^ ^]^ These results further demonstrated that the MOFs membrane sensor array chip could stably detect ACN‐H_2_O crude extract solutions and high‐temperature extraction environments directly, without the need for complex pretreatment procedures. Moreover, we also used commercial immunochromatographic test strips to detect OTC, TMP, and CIP (1 µg mL^−1^) in both the corresponding buffer solution and ACN‐H_2_O. Figure 5e showed that the test strips failed to develop color properly in ACN‐H_2_O, due to the aggregation of gold nanoparticle probes caused by the organic agents, which impeded further flow. It highlighted the superior structural and detection stability of the fluorescent MOFs membrane array sensor, which relied on molecular recognition. The obtained MOFs membrane sensor array remained at almost its original ED values during 30 days of storage at 25 °C (Figure 5f), indicating great processability due to the excellent physical properties of MOFs materials.

Finally, the recovery tests with spiked chicken samples were performed to demonstrate the multiple detection and reliability of MOFs membrane array in real samples. Chicken samples were subjected to a one‐step extraction using an ACN‐H_2_O solution. After precipitation, the supernatant was collected and mixed with antibiotics at different concentrations to prepare mixed antibiotic solutions with final concentrations of 0.1 and 0.75 µg mL^−1^. Subsequently, the MOFs membrane sensor array chip enabled the simultaneous quantitative detection of three antibiotics (OTC, TMP, and CIP) in the mixed solution through a single filtration process lasting 10 min. As shown in **Table** **1**, the MOFs membrane sensor array chip achieved satisfactory detection results with recoveries ranging from 84.6% to 121.7%, indicating a good accuracy for the simultaneous determination of mixed antibiotics samples in agricultural and environmental samples.

**Table 1:** Simultaneous quantitative determination of mixed antibiotics in chicken samples based on MOFs membrane sensor array.

Mixed antibiotics components	Added [µg mL^−1^]	Detected [µg mL^−1^]	Recovery [%]
OTC	0.1	0.08±0.02	84.60±17.30
	0.75	0.81±0.45	107.60±30.10
TMP	0.1	0.09±0.03	89.90±25.40
	0.75	0.65±0.18	86.80±11.80
CIP	0.1	0.12±0.01	121.70±8.30
	0.75	0.65±0.001	87.20±0.09

## 3 Conclusion

In summary, we have proposed a fluorescent MOFs hybrid membrane sensor array to develop a one‐to‐one recognition and direct quantification method for the simultaneous detection of multiple antibiotics without extensive data processing. Representative MOFs (PCN‐128, Eu‐MOFs, and Tb‐MOFs) were designed based on the chemical structural characteristics of different antibiotics. These MOFs exhibited unique fluorescence responses to target antibiotics (OTC, TMP, and CIP) through electron transfer/energy transfer mechanisms, addressing the challenge of low selectivity in chemical recognition. The single MOFs hybrid membranes, prepared via vacuum‐assisted filtration and microwave‐assisted solvothermal methods, leveraged the effective enrichment of highly specific MOFs recognition elements. These membranes exhibited one‐to‐one high selectivity for antibiotic detection and significantly reduced the detection limits. By combining the inherent porosity of the filter membrane with the high adsorption capacity, specificity, and responsiveness of MOFs, the fluorescence membrane sensor array enabled direct, quantitative, and simultaneous detection of OTC, TMP, and CIP in mixed solutions at ppb levels during filtration. This approach eliminates the need for separate recognition, separation, labeling, and signal generation steps, streamlining the entire detection process into a single procedure that can be completed within 10 min. The MOFs membrane sensor array also presented anti‐interference ability and robustness. Remarkably, the fluorescent MOFs membrane sensor array could be facilely integrated into a home‐made smartphone‐based portable device and enable direct quantitative detection of multiple antibiotics in chicken samples based on the custom‐developed image analysis applet with one‐step extraction and a single filtration process. The fluorescent MOFs membrane array may be developed as high‐throughput and in‐field tools for antibiotics in agriculture and food.

## 4 Experimental Section

### Materials

Zirconium chloride (ZrCl_4_, 99.5%), terbium nitrate hexahydrate (Tb(NO_3_)_3_·6H_2_O, 99.9%), europium nitrate hexahydrate (Eu(NO_3_)_3_·6H_2_O, 99.9%), 4,4′,4″‐(1,3,5‐triazine‐2,4,6‐triyl)tribenzoic acid (H_3_TATB, 97.0%), 5′‐monophosphate monohydrate (5′‐AMP, 99.0%), Tris(hydroxymethyl)aminomethane (Tris, 99%), and cetyltrimethylammonium bromide (CTAB, 99.0%) were purchased from Aladdin Industrial Corporation (Shanghai, China). Ammonium hydroxide (NH_4_OH, 25.0%–28.0%), N, N‐dimethylformamide (DMF, 99.0%), isooctane (99.9%), methanol (99.9%), ethanol (99.9%), nitric acid (HNO_3_, 99.9%) and acetonitrile (ACN, 99.9%) were purchased from Sinopharm Chemical Reagent Co. Ltd. (Shanghai, China). 4′,4″,4′″,4″″‐(ethene‐1,1,2,2‐tetrayl)tetrabiphenyl‐4‐carboxylic acid (H_4_ETTC, 98%) was purchased from Academy of Sciences‐Yanshen Technology Co., Ltd. (Jilin, China) 1‐Hexanol (98.0%) was purchased from Bangyi Chemical Co., Ltd (Hangzhou, China). OTC, TMP, CIP, LEV, CTC, CAP, CEF, ENR, FZD, GEN, KAN, LIN, MNZ, NFT, NFZ, NOR, PEN, RTD, SDZ, TET, Lys, E, Asp, Gly, Pro, Glu, Gal, Fru, CdCl_2_ and Pb(CH_3_COO)_2_·3H_2_O were purchased from Aladdin Industrial Corporation (Shanghai, China). Ultrapure water was used throughout the Milli‐Q ultrapure water system (18.2 MΩ cm, Millipore, Billerica, MA, USA). All materials were used as received without further purification.

### Characterization

The morphologies of the three kinds of MOFs, membrane, and hybrid membrane were all characterized by investigation using a field‐emission SEM (SU8010, HITACHI). Elemental component analysis was performed using an energy‐dispersive X‐ray spectroscope (SU8010, HITACHI). The structure of all samples, including three kinds of MOFs, membranes, and prepared membranes, was analyzed using an X‐ray diffractometer (D8 ADVANCE, Bruker, Germany). Water contact angles were measured by an optical contact angle device (OSA200‐T, NBSI, China). The surface area data of three kinds of MOFs was measured on Quantachrome Instruments at 77 K (surface area and pore size analyzer NOVA touch LX4). TGA of three kinds of MOFs was performed on TGA 2 (METTLER TOLEDO, Switzerland) in the temperature range from 30 to 800 °C with a heating rate of 10 °C min^−1^ under an N_2_ atmosphere. FT–IR of three kinds of MOFs, membranes, and prepared membranes was performed using an AVATAR 370 spectrometer (Thermo Nicolet, USA). Fabrication was collected on a household microwave oven, model G80F20CN1L‐DG (W0) (Guangdong, China), with a maximum microwave output power of 800 W. All fluorescence experiments of three kinds of MOFs were conducted on a Synergy H1 hybrid multi‐mode microplate reader (BioTek Instruments Inc., USA). UV–vis absorption spectra of powder samples were measured on an Agilent 8453 UV–vis spectrophotometer (Agilent, USA).

### Synthesis of PCN‐128

ZrCl_4_ (120.0 mg) and H_4_ETTC (60.0 mg) were dissolved in 8 mL DMF solution. After uniform dispersion, the mixture was sonicated for 10 min and heated at 120 °C for 48 h. After being cooled to room temperature, the faint yellow powder was collected by centrifugation (6000 rpm for 10 min), and washed with DMF, water, and ethanol, consecutively. The product dried at 60 °C for 12 h for further use.

### Synthesis of Eu‐MOFs

Eu‐MOFs were synthesized using a novel microwave‐assisted method. First, CTAB (0.455 g), 1‐hexanol (2.355 mL), and isooctane (24.35 mL) were sonicated to obtain two identical reaction solutions, which were then mixed thoroughly on a vortex mixer for 30 min. Subsequently, H_3_TATB (15.89 mg) was dispersed in an ammonium hydroxide solution (720 µL) using ultrasound to obtain a clear solution, while Eu(NO_3_)_3_·6H_2_O (32 mg) was dispersed in a water solution (720 µL). These solutions were separately added to the reaction solutions mentioned earlier and mixed for another 2 h. The precursor solutions containing H_3_TATB and Eu(NO_3_)_3_·6H_2_O were then transferred to a polytetrafluoroethylene reaction vessel and placed in a microwave oven with a power setting of 160 W for 15 min. The resulting precipitate was washed with methanol, ethanol, and water at 6000 rpm for 10 min and then dried at 60 °C for 12 h before collection for further use.

### Synthesis of Tb‐MOFs

First, 0.25 mmol Tb(NO_3_)_3_·6H_2_O was uniformly dispersed in 11 mL of ultrapure water to form a homogeneous suspension. Subsequently, under constant stirring, 15 mL of Tris buffer solution (0.1 m, pH = 7.4) containing 0.25 mmol 5′‐AMP was added to the suspension, and the reaction was stirred at room temperature for 2 h. The resulting precipitate was washed with methanol, ethanol, and water at 10 000 rpm for 10 min each and then dried at 60 °C for 12 h before collection for further use.

### Preparation of Nylon@Eu‐MOFs and Nylon@Tb‐MOFs

Nylon@Eu‐MOFs and Nylon@Tb‐MOFs were prepared via a simple vacuum filtration process. Eu‐MOFs and Tb‐MOFs were dispersed in ethanol using ultrasound to prepare homogeneous suspensions. To regulate the loading number of MOFs on each hybrid membrane for optimal detection performance, different amounts of Eu‐MOFs (20, 60, 100, 144, 200 µg) and Tb‐MOFs (5, 10, 25, 50, 100 µg) were filtered through the vacuum‐assisted self‐assembly process onto 13 mm commercial Nylon membrane substrates. The resulting hybrid membranes were immersed in ethanol for 5 min, followed by drying at 60 °C for 10 min for further use.

### Preparation of Nylon@PCN‐128

Nylon@PCN‐128 was synthesized via the microwave‐assisted hydrothermal method. The Nylon membrane was immersed in 4 mL of precursor mixture (0.15 mg mL^−1^ ZrCl_4_, 0.075 mg mL^−1^ H_4_ETTC in DMF solution) for 10 min. The reaction was then carried out at different microwave powers (80–640 W) for 10 min. Finally, the Nylon@PCN‐128 was washed three times with DMF and ultrapure water and dried at 60 °C for 10 min.

### Preparation of Fluorescent MOFs Membrane Sensor Array

The central regions of three types of 13 mm Nylon@MOFs and Nylon membranes were cut into 9 mm circles and then assembled into a portable membrane array using brackets processed by a numerical control machine.

### Antibiotics Sensing Based on MOFs

As for the PCN‐128‐based sensor, PCN‐128 was dispersed in H_2_O with sonication and then mixed with the OTC solution. The fluorescence emission spectra of PCN‐128 in supernatants were collected after the incubation with different concentrations of OTC. The fluorescent emission spectrum and the corresponding QR were collected to establish the linear relationship between QR and OTC for detection. QR value was calculated as follows: QR =(F_0_−F)/F_0_, where F_0_ and F was the intensity of fluorescence without and with antibiotics, respectively. The detection was based on the fluorescence enhancement of the Eu‐MOFs, and Tb‐MOFs toward TMP and CIP, respectively. The ER value was calculated as follows: ER =(F−F_0_)/F_0_, where F_0_ and F were the intensity of fluorescence without and with antibiotics, respectively. The remaining operational steps were identical to those of PCN‐128.

### Antibiotics Sensing Based on Single MOFs Membrane

The single MOFs membranes were washed with extensive water and ethyl alcohol to remove residuary phosphate saline and unstable MOFs before the detection process. The quantitative detection capability of the hybrid membrane could also be determined by the ED value after the reaction. ED =$\sqrt{{\left(R-R_{0}\right)}^{2}+{\left(G-G_{0}\right)}^{2}+{\left(B-B_{0}\right)}^{2}}$, R_0_, B_0_, and G_0_ represented the color of fluorescent images before the reaction). A larger ED value represents a stronger response to antibiotics.

### Sample Extraction and Pretreatment

Commercial chicken breast samples were purchased from local markets and treated according to the following procedures. First, the chicken breast meat was grinded to get homogenate. Then, 5.0 g of chicken meat homogenate was weighed and transferred into a polypropylene tube containing 10 mL of ACN‐H_2_O, the sample was extracted ultrasonically for 5 min. Finally, the mixture solution was allowed to settle naturally for several minutes, and the supernatant was collected to obtain the crude extract of the chicken breast sample.

### Simultaneous Quantification by the Smartphone‐Based 3D‐Printed Device

The smartphone‐based 3D‐printed device consisted of a smartphone, 3D‐printed top and bottom covers, strip holder, LED, polymer lithium‐ion battery, and transformer. The device has an overall dimension of ≈150 mm × 80 mm × 85 mm. The printing material was black to prevent light penetration from the surrounding environment and minimize light leakage. The excitation lights were provided by a UV LED (365 nm) powered by a polymer lithium‐ion battery, which was fixed at four corners of the instrument and illuminated the membrane sensor array at an incidence angle of 45°. Finally, the transmitted fluorescence images of MOFs membrane and sensor array were collected by CMOS sensor of a smartphone and calculate the concentrations of different antibiotics.

## Conflict of Interest

The authors declare no conflict of interest.

## Supporting information



Supporting Information

## Data Availability

The data that support the findings of this study are available from the corresponding author upon reasonable request.

## References

[1] D. Li , H. Xing , Y. Yang , S. Su , W. Li , M. Hu , Inorg. Chem. 2024, 63, 19167. 39352230 10.1021/acs.inorgchem.4c02736

[2] K. H. Lee , H. Jang , Y. S. Kim , C. H. Lee , S. H. Cho , M. Kim , H. Son , K. B. Bae , D. V. Dao , Y. S. Jung , I. H. Lee , Adv. Sci. 2021, 8, 2100640. 10.1002/advs.202100640 PMC8498916 34363354

[3] Z. Zhang , H. Zhang , D. Tian , A. Phan , M. Seididamyeh , M. Alanazi , Z. P. Xu , Y. Sultanbawa , R. Zhang , Coord. Chem. Rev. 2024, 498, 215455.

[4] J. A. Lewnard , N. C. Lo , N. Arinaminpathy , I. Frost , R. Laxminarayan , Nature 2020, 581, 94. 32376956 10.1038/s41586-020-2238-4 PMC7332418

[5] F. Wong , E. J. Zheng , J. A. Valeri , N. M. Donghia , M. N. Anahtar , S. Omori , A. Li , A. Cubillos‐Ruiz , A. Krishnan , W. Jin , A. L. Manson , J. Friedrichs , R. Helbig , B. Hajian , D. K. Fiejtek , F. F. Wagner , H. H. Soutter , A. M. Earl , J. M. Stokes , L. D. Renner , J. J. Collins , Nature 2024, 626, 177. 38123686 10.1038/s41586-023-06887-8 PMC10866013

[6] Q.‐Q. Li , M.‐J. Wen , Y.‐S. Zhang , Z.‐S. Guo , X. Bai , J.‐X. Song , P. Liu , Y.‐Y. Wang , J.‐L. Li , J. Hazard. Mater. 2022, 423, 127132. 34537652 10.1016/j.jhazmat.2021.127132

[7] L. Yang , Y. Shen , J. Jiang , X. Wang , D. Shao , M. M. C. Lam , K. E. Holt , B. Shao , C. Wu , J. Shen , T. R. Walsh , S. Schwarz , Y. Wang , Z. Shen , Nat. Food 2022, 3, 197. 37117646 10.1038/s43016-022-00470-6

[8] M. Majdinasab , R. K. Mishra , X. Tang , J. L. Marty , TrAC, Trends Anal. Chem. 2020, 127, 115883.

[9] K. Birader , P. Kumar , Y. Tammineni , J. A. Barla , S. Reddy , P. Suman , Food Chem. 2021, 356, 129659. 33812186 10.1016/j.foodchem.2021.129659

[10] Y. Zhang , Y. Hu , S. Deng , Z. Yuan , C. Li , Y. Lu , Q. He , M. Zhou , R. Deng , J. Agric. Food Chem. 2020, 68, 2554. 32027503 10.1021/acs.jafc.0c00141

[11] Z. Li , J. R. Askim , K. S. Suslick , Chem. Rev. 2019, 119, 231. 30207700 10.1021/acs.chemrev.8b00226

[12] Y. Zhang , T. Wang , H. Guo , X. Gao , Y. Yan , X. Zhou , M. Zhao , H. Qin , Y. Liu , Biosens. Bioelectron. 2023, 231, 115266. 37058957 10.1016/j.bios.2023.115266

[13] X. Wang , H. Qi , Y. Shao , M. Zhao , H. Chen , Y. Chen , Y. Ying , Y. Wang , Adv. Sci. 2024, 11, 2400207. 10.1002/advs.202400207 PMC11220709 38655847

[14] Y. Tian , L. Zhang , H. Wang , W. Ji , Z. Zhang , Y. Zhang , Z. Yang , Z. Cao , S. Zhang , J. Chang , ACS Sens. 2019, 4, 1873. 31259533 10.1021/acssensors.9b00752

[15] F. Li , M. Zhu , Z. Li , N. Shen , H. Peng , B. Li , J. He , Talanta 2024, 269, 125446. 38043343 10.1016/j.talanta.2023.125446

[16] H. Che , X. Tian , F. Guo , Y. Nie , C. Dai , Y. Li , L. Lu , Anal. Chem. 2023, 95, 12550. 37550863 10.1021/acs.analchem.3c02911

[17] X. Tan , Y. Liang , Y. Ye , Z. Liu , J. Meng , F. Li , Anal. Chem. 2022, 94, 829. 34978809 10.1021/acs.analchem.1c03508

[18] M. Kalaj , K. C. Bentz , S. Ayala Jr. , J. M. Palomba , K. S. Barcus , Y. Katayama , S. M. Cohen , Chem. Rev. 2020, 120, 8267. 31895556 10.1021/acs.chemrev.9b00575

[19] L. J. Small , S. E. Henkelis , D. X. Rademacher , M. E. Schindelholz , J. L. Krumhansl , D. J. Vogel , T. M. Nenoff , Adv. Funct. Mater. 2020, 30, 2006598.

[20] L. Kong , C. Yu , Y. Chen , Z. Zhu , L. Jiang , Small 2024, 20, 2407021. 10.1002/smll.202407021 39444085

[21] L. Zhang , Y. Sun , Z. Zhang , Y. Shen , Y. Li , T. Ma , Q. Zhang , Y. Ying , Y. Fu , Biosens. Bioelectron. 2022, 216, 114659. 36095979 10.1016/j.bios.2022.114659

[22] X. Ma , Y. Li , J. Zhang , T. Ma , L. Zhang , Y. Chen , Y. Ying , Y. Fu , ACS Appl. Mater. Interfaces 2023, 15, 27034. 37232292 10.1021/acsami.3c01094

[23] Z. Li , X. Xu , H. Quan , J. Zhang , Q. Zhang , Y. Fu , Y. Ying , Y. Li , Chem. Eng. J. 2021, 410, 128268.

[24] X. Fang , X. Wang , Y. Li , Q. Li , S. Mao , Anal. Chem. 2023, 95, 2436. 36650048 10.1021/acs.analchem.2c04613

[25] K. Yi , L. Zhang , Food Chem. 2021, 354, 129584. 33761339 10.1016/j.foodchem.2021.129584

[26] L. Zhang , Y. Sun , L. Peng , W. Fang , Q. Huang , J. Zhang , Z. Zhang , H. Li , Y. Liu , Y. Ying , Y. Fu , Adv. Sci. 2023, 10, 2204702. 10.1002/advs.202204702 PMC9839836 36412067

[27] H.‐R. Fu , L.‐B. Yan , N.‐T. Wu , L.‐F. Ma , S.‐Q. Zang , J. Mater. Chem. A 2018, 6, 9183.

[28] G. Qin , J. Wang , L. Li , F. Yuan , Q. Zha , W. Bai , Y. Ni , Talanta 2021, 221, 121421. 33076058 10.1016/j.talanta.2020.121421

[29] Y. Zhao , Q. Wang , H. Wang , H. Zhangsun , X. Sun , T. Bu , Y. Liu , W. Wang , Z. Xu , L. Wang , Sens. Actuators B: Chem. 2021, 334, 129610.

[30] W. Hu , N. Wu , D. Li , Y. Yang , S. Qie , S. Su , R. Xu , W. Li , M. Hu , J. Mater. Chem. C 2025, 13, 592.

[31] L. Liu , Q. Chen , J. Lv , Y. Li , K. Wang , J.‐R. Li , Inorg. Chem. 2022, 61, 8015. 35544341 10.1021/acs.inorgchem.2c00754

[32] H. Wang , X. Qian , X. An , Carbohydr. Polym. 2022, 287, 119337. 35422301 10.1016/j.carbpol.2022.119337

[33] S. Su , D. Li , Y. Yang , R. Xu , W. Hu , W. Li , M. Hu , Appl. Organomet. Chem. 2024, 38, 7524.

[34] Y. Zhou , Q. Yang , D. Zhang , N. Gan , Q. Li , J. Cuan , Sens. Actuators B: Chem. 2018, 262, 137.

[35] Y. Li , J. Wang , Z. Huang , C. Qian , Y. Tian , Y. Duan , J. Environ. Chem. Eng. 2021, 9, 106012.

[36] M. Jian , X. Sun , S. Li , H. Wang , H. Zhang , X. Li , Y. He , Z. Wang , Anal. Chem. 2024, 96, 7353. 38690857 10.1021/acs.analchem.4c00266

[37] S. Wang , Z. Wang , L. Zhang , Y. Xu , J. Xiong , H. Zhang , Z. He , Y. Zheng , H. Jiang , J. Shen , Food Chem. 2022, 374, 131712. 34920407 10.1016/j.foodchem.2021.131712

