# Supplementary material for: Tailored Fluorescent Metal–Organic Frameworks Hybrid Membrane Sensor Arrays: Simultaneous and Selective Quantification of Multiple Antibiotics
Source: Adv Sci (Weinh). 2025 Apr 26;12(27):2502452. doi: 10.1002/advs.202502452 (PMC12279192; doi:10.1002/advs.202502452)
Supplement: Supplementary file 1 — Supporting Information [file ADVS-12-2502452-s001.docx]

Supporting Information

**Tailored Fluorescent Metal-Organic Frameworks Hybrid Membrane Sensor Arrays: Simultaneous and Selective Quantification of Multiple Antibiotics**

Tongtong Ma, Qiao Huang, Lei Yuan, Shugang Yan, Yalin Mo, Yibin Ying, Yingchun Fu*, Jinming Pan*

College of Biosystems Engineering and Food Science, Zhejiang Key Laboratory of Intelligent Sensing and Robotics for Agriculture, Zhejiang University, Hangzhou 310058, China;

*Correspondence to: Prof. Yingchun Fu, Prof. Jinming Pan

E-mail: [ycfu@zju.edu.cn](mailto:ycfu@zju.edu.cn), panhouse@zju.edu.cn


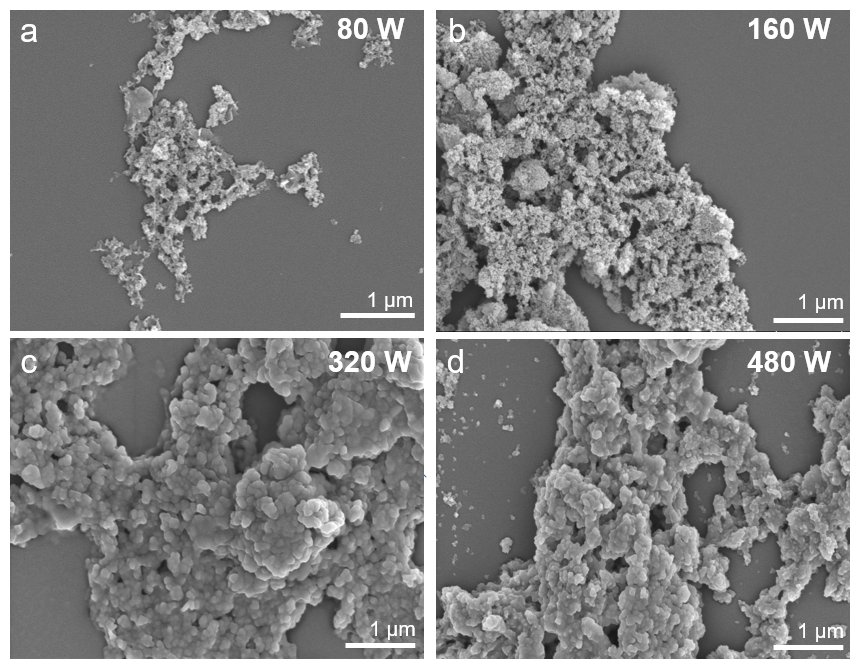


**Figure S1.** SEM images of Eu-MOFs synthesized under microwave power of (a) 80 W, (b) 160 W, (c) 320 W, (d) 480 W.

**
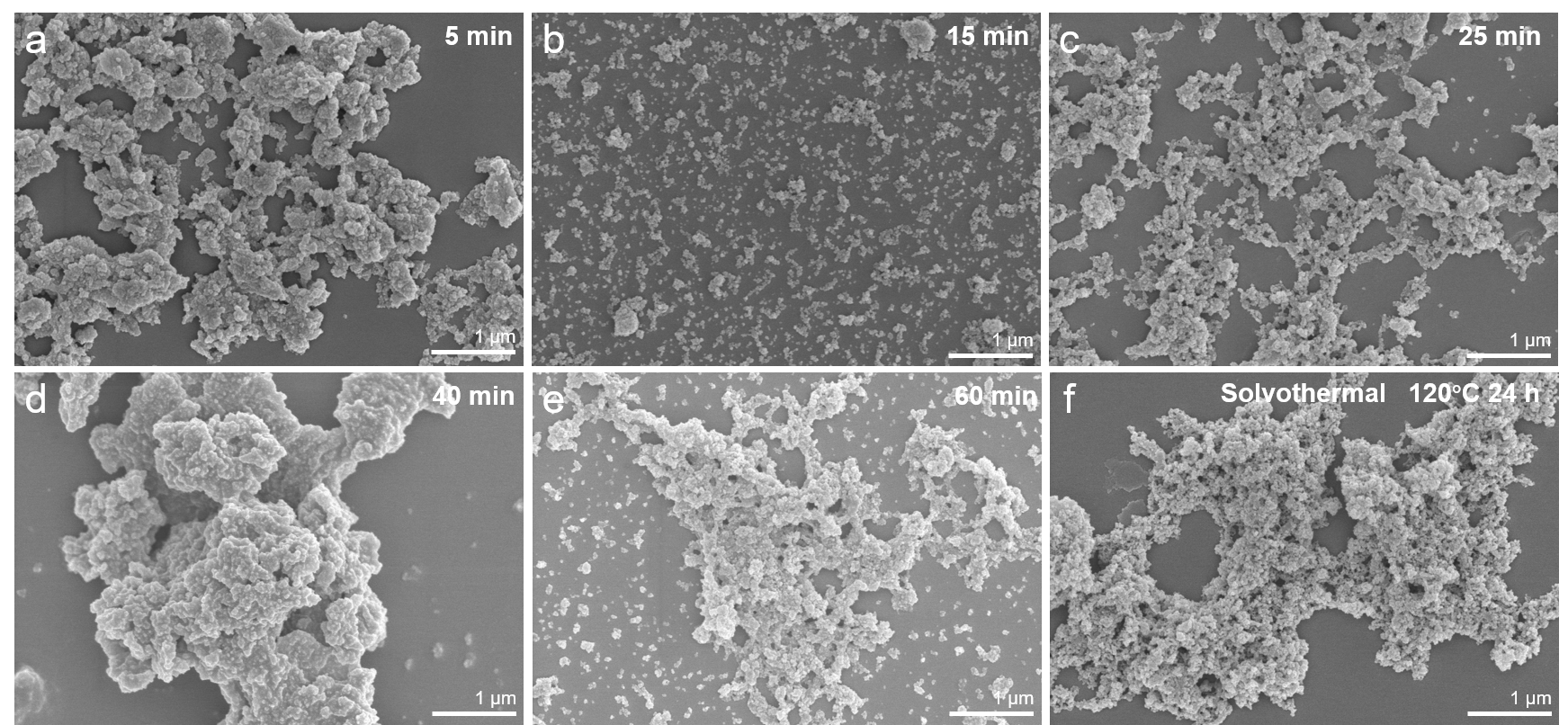
**

**Figure S2.** SEM images of Eu-MOFs synthesized under reaction time of (a) 5 min, (b) 15 min, (c) 25 min, (d) 40 min, (e) 60 min, at 160 W, and (f) solvothermal reaction of 120 °C for 24 h.

Figure S1, S2 showed that as the power increased and reaction time lengthened, Eu-MOFs tended to aggregate and grow, resulting in a reduced specific surface area. Moreover, the synthetic Eu-MOFs exhibited a morphology similar to those synthesized by the conventional hydrothermal method, but with significantly improved efficiency and enhanced the dispersion of particles. Therefore, considering the synthesis efficiency, 160 W of the microwave power, 15 min of reaction time was chosen as the optimized parameters.

**
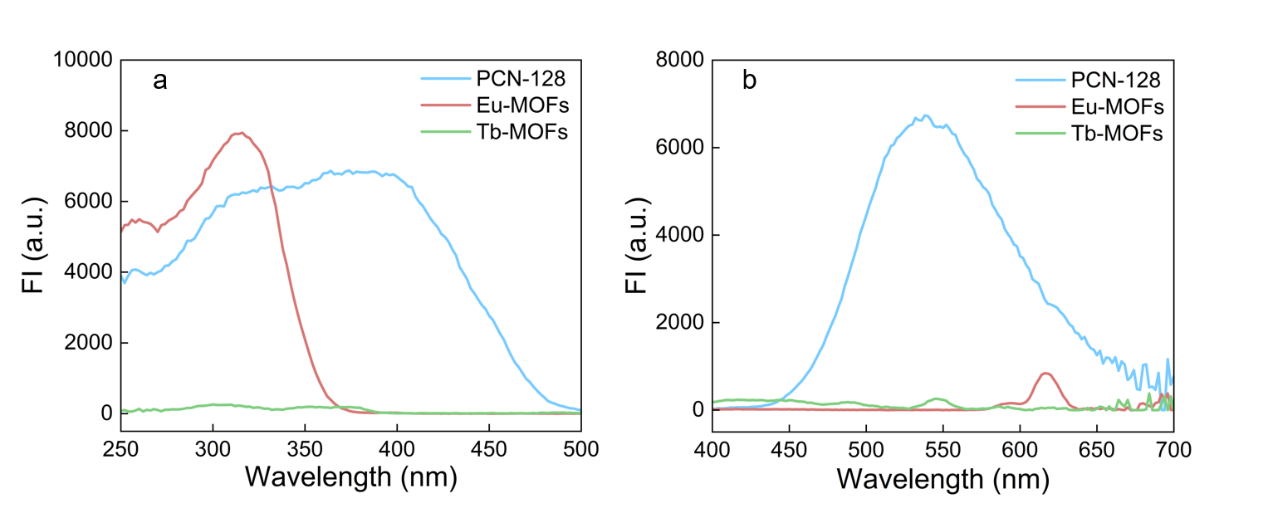
**

**Figure S3.** (a) Fluorescent excitation and (b) emission spectra of PCN-128, Eu-MOFs and Tb-MOFs.

The maximum excitation wavelengths of Eu-MOFs and Tb-MOFs were 320 nm and 360 nm, respectively, whereas PCN-128 exhibited a broad excitation band between 300-400 nm, primarily attributed to the fluorescent excitation properties of the organic ligand of PCN-128 (H_4_ETTC). The maximum emission wavelengths of PCN-128, Eu-MOFs and Tb-MOFs were 546 nm, 620 nm and 546 nm, respectively.

**
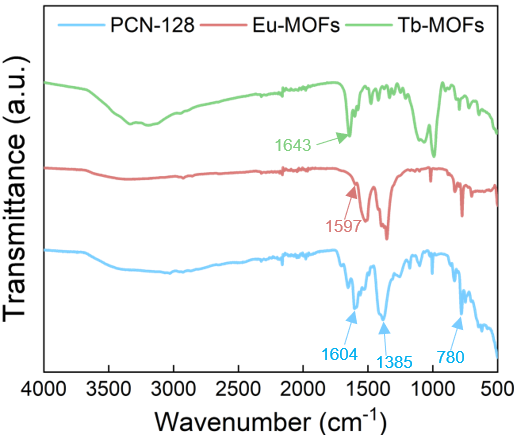
**

**Figure S4.** FT-IR spectra of PCN-128, Eu-MOFs and Tb-MOFs.

In the case of PCN-128, two characteristic peaks of FT-IR were observed at 1385 cm^-1^ and 1604 cm^-1^, which were attributed to the carboxyl vibration of PCN-128. The peak at 780 cm^-1^, attributed to the stretching vibration of the Zr-O bond in the Zr-oxo cluster, confirmed the successful synthesis of PCN-128 ^[1]^. For Eu-MOFs, the peaks at 1208 cm^-1^, 2521 cm^-1^, 2651 cm^-1^, and a broad peak at 3360 cm^-1^ are all belonged to the stretching vibration of O-H in carboxyl of the ligand (H_3_TATB, 4,4’,4’’-triazine-2,4,6-triyltribenzoate, Figure S5) ^[2]^. The disappearance of the four peaks and the bathochromic shift of the peak at 1582 cm^-1^ are attributed to the successful connection of Eu^3+^ with H_3_TATB. Besides, the FT-IR spectra comparison between Tb-MOFs and 5’-AMP revealed a peak at 1643 cm^-1^ corresponding to the scissoring vibration of -NH_2_, confirming the successful preparation of Tb-MOFs ^[3]^.

**
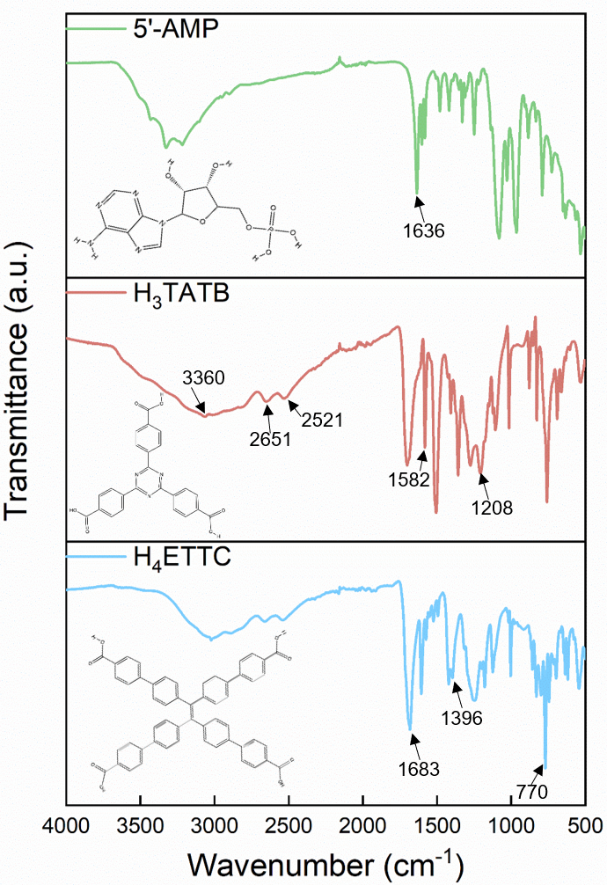
**

**Figure S5.** FT-IR spectra of the different organic ligands (H_4_ETTC, H_3_TATB, 5’-AMP) of three kinds of MOFs.

**
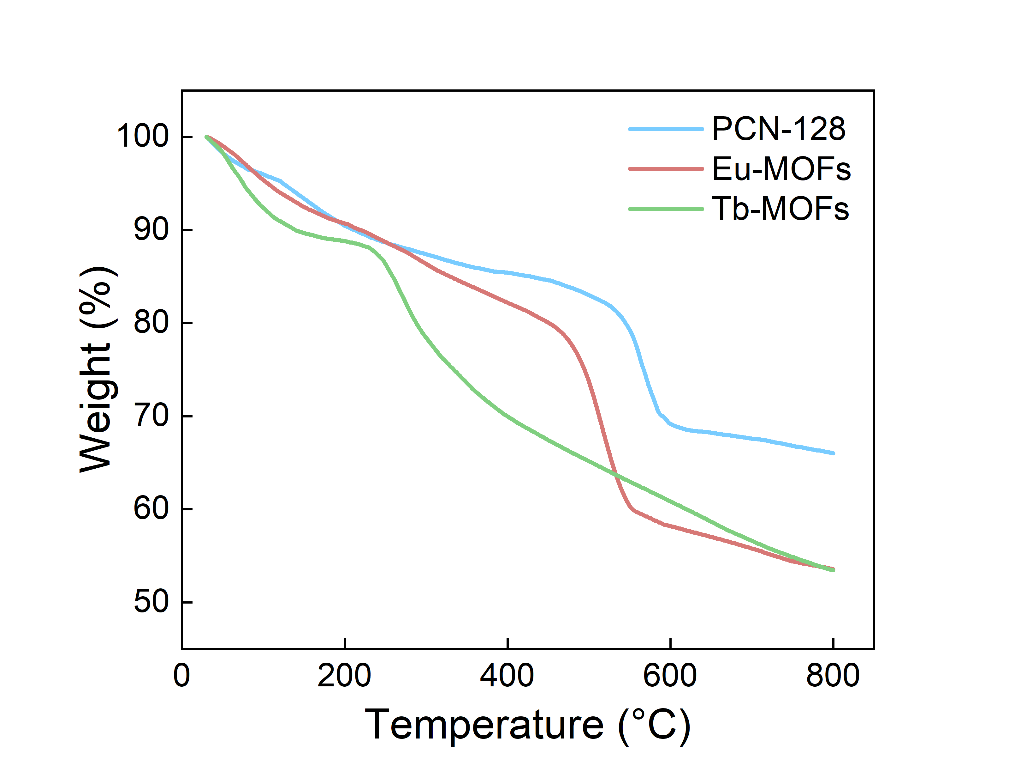
**

**Figure S6.** TGA curves of PCN-128, Eu-MOFs and Tb-MOFs.

TGA indicated that PCN-128, Eu-MOFs and Tb-MOFs possessed excellent thermal stability within a high decomposition temperature up to 400°C and 250°C, which can be attributed to the strong bonding between the metal ions and the organic ligand linkers and carboxylates.

**
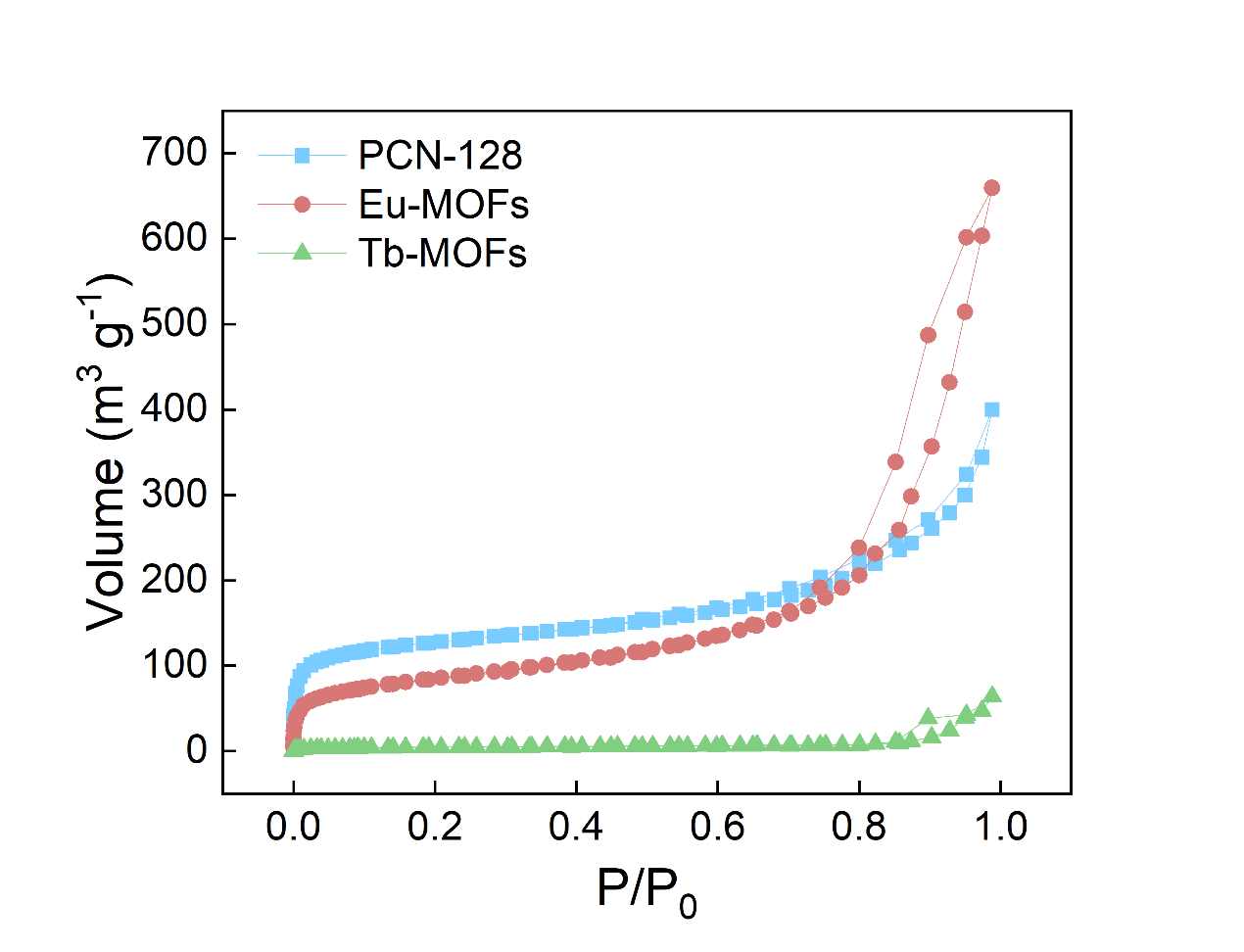
**

**Figure S7.** The N_2_ adsorption-desorption isotherms of PCN-128, Eu-MOFs and Tb-MOFs.

The N_2_ adsorption-desorption isotherms of the three MOFs all displayed Type I isotherms, indicating that they are microporous materials with excellent adsorption properties. The specific surface area of PCN-128 and Eu-MOFs are 471.1 m^3^ g^-1^ and 303.7 m^3^ g^-1^, respectively, while Tb-MOFs is only 17.1 m^3^ g^-1^, primarily due to its gel-like morphology, which reduced its porosity.


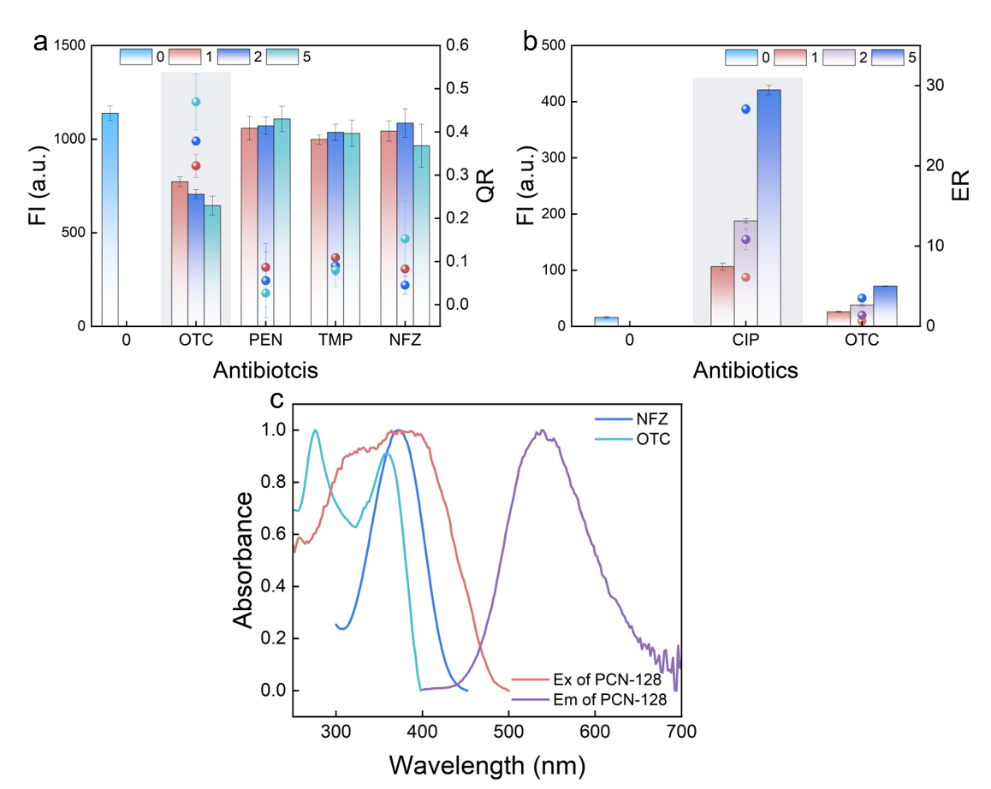


**Figure S8.** (a) FI and QR of PCN-128 after reacting with 1 μg mL^-1^, 2 μg mL^-1^, and 5 μg mL^-1^ of OTC, PEN, TMP, and NFZ. (b) FI and ER of Tb-MOFs after reacting with 1 μg mL^-1^, 2 μg mL^-1^, and 5 μg mL^-1^ of CIP and OTC. (c) Excitation and emission spectra of PCN-128 and UV-vis spectrum of NFZ and OTC.

At the same concentration, PCN-128 exhibited a significantly higher fluorescence quenching effect toward OTC. As the OTC concentration increased from 1 µg mL^-1^ to 5 µg mL^-1^, the QR of PCN-128 raised from 0.321±0.027 to 0.469±0.065. However, the QRs for PEN and TMP are only 0.027±0.033 and 0.08±0.038, even at a high concentration of 5 µg mL^-1^, which we consider negligible. Additionally, at lower concentrations (1 µg mL^-1^ and 2 µg mL^-1^), NFZ did not cause significant fluorescence quenching, with QR of 0.083±0.004 and 0.045±0.021, respectively. However, at a higher concentration (5 µg mL^-1^), NFZ exhibited a relatively higher QR of 0.153±0.088. This may be attributed to its ultraviolet absorption wavelength (300-400 nm), which is similar to that of OTC (250-400 nm), and both overlap with the fluorescence excitation wavelength of PCN-128, leading to an IFE effect that influences the FI of PCN-128. Indeed, this overlap between tetracycline antibiotics like OTC and nitro-based antibiotics like NFZ is very common in MOFs fluorescence detection applications and has always been a challenging issue in the detection applications of chemical recognition elements ^[1, 4-5]^. Fortunately, the interference response of PCN-128 to NFZ is relatively low, representing one of the best specificities among existing methods.

Tb-MOFs primarily exhibit significant fluorescence enhancement in response to fluoroquinolone antibiotics, represented by CIP. Moreover, to verify the response of Tb-MOFs to tetracycline antibiotics, we selected OTC as the interfering antibiotic and compared it with CIP. As the concentration increased from 1 µg mL^-1^ to 5 µg mL^-1^, the ER increased from 6.1±0.424 to 27.067±0.566, while OTC only increased from 0.637±0.18 to 3.511±0.444, which was only 10%-13% of that of CIP, and can be considered negligible based on literature references ^[6]^.

In summary, although PCN-128 and Tb-MOFs cannot completely eliminate interference from other classes of antibiotics, their recognition differences compared to the target antibiotics (OTC and fluoroquinolones) are significant.


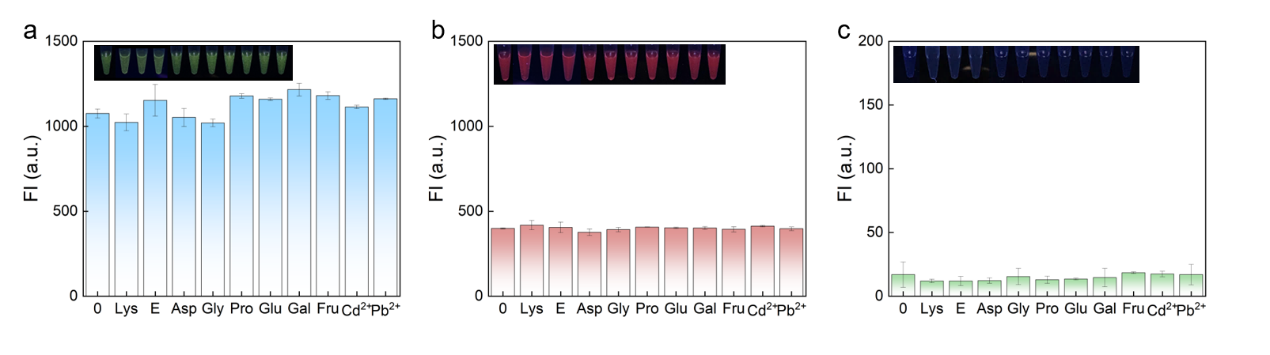


**Figure S9.** Fluorescent responses of (a) PCN-128, (b) Eu-MOFs, and (c) Tb-MOFs to different types of amino acids, sugars, and heavy metal ions.

Among them, only Lys Asp, and Gly induced a minimal degree of fluorescence quenching in PCN-128, with quenching rates of 0.057±0.019, 0.028±0.076 and 0.059±0.006, respectively. Under the same conditions, OTC caused a QR of 0.375±0.081 in PCN-128. Therefore, the quenching effects of these amino acids can be considered negligible. Moreover, all amino acids, sugars, and heavy metal ions did not cause fluorescence enhancement or quenching in Eu-MOFs and Tb-MOFs. These results all demonstrate the high selectivity of the PCN-128, Eu-MOFs, and Tb-MOFs.

**
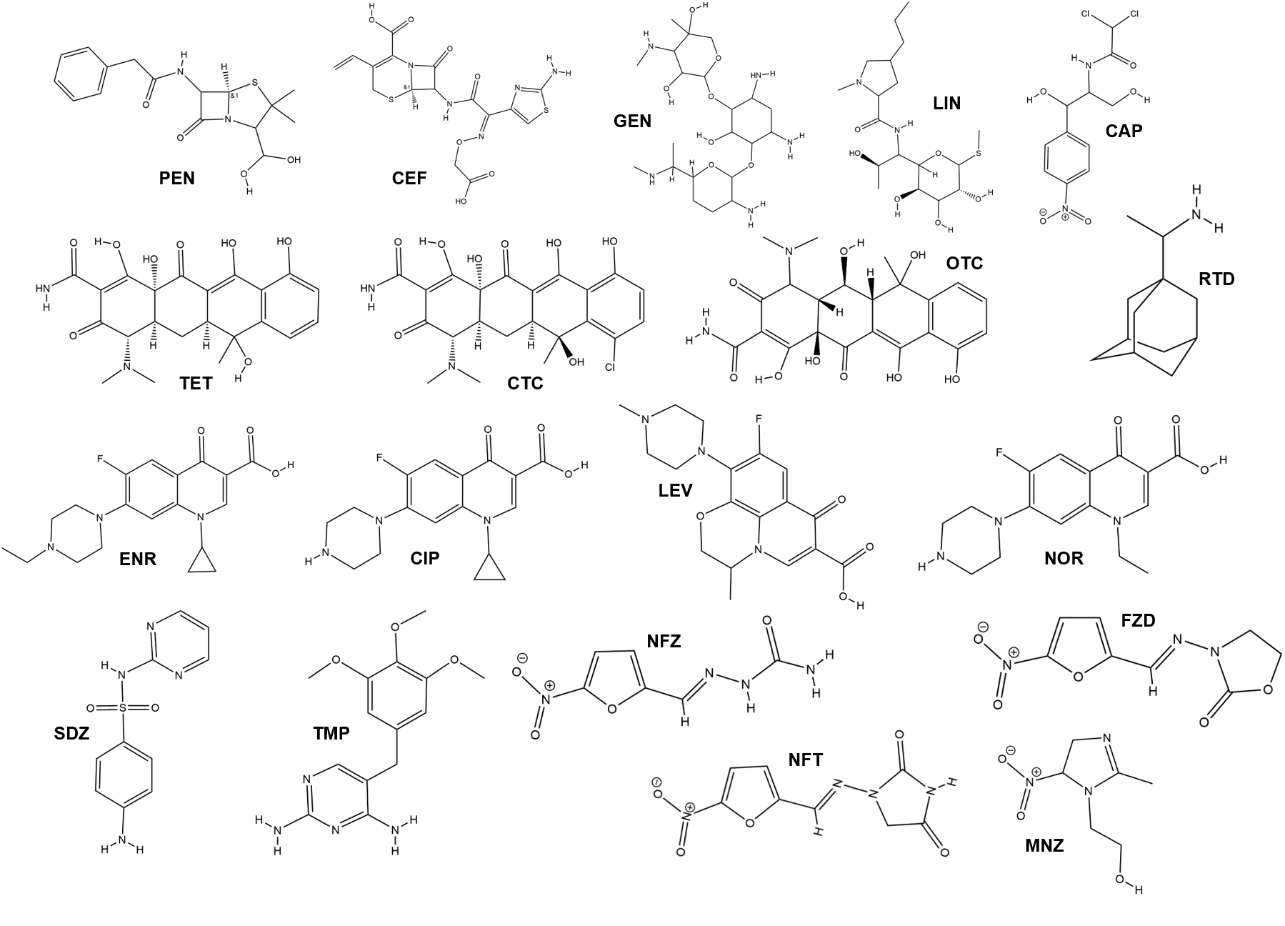
**

**Figure S10.** The structural formula of antibiotics.

The PCN-128 exhibited a significant decrease of fluorescence intensity after reacting with OTC, mainly due to the strong adsorption of OTC and electron transfer. This effect was further enhanced by the greater number of -OH groups in OTC, which could interact with the terminal -OH/H_2_O groups at the Zr_6_ nodes of PCN-128 through solvent-assisted ligand incorporation ^[1]^.

**
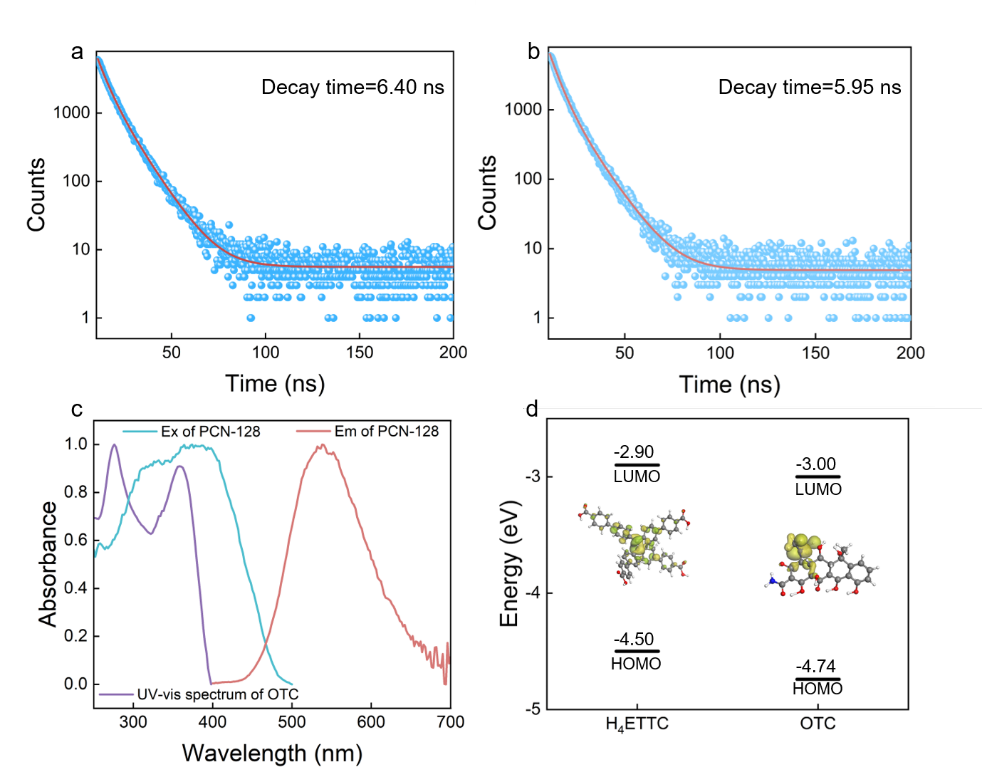
**

**Figure S11**. (a) Fluorescence lifetime curve for PCN-128. (b) Fluorescence life fitting curve of PCN-128 soaked in OTC solution. (c) Excitation and emission spectra of PCN-128 and UV-vis spectrum of OTC. (d) The HOMO and LUMO energies for organic ligand (H_4_ETTC) and OTC.

The fluorescence lifetime of PCN-128 decreased from 6.40 ns to 5.95 ns after reacting with OTC, indicating the presence of a dynamic quenching process. Secondly, the UV-vis spectrum of OTC shows minimal overlap with the emission spectrum of PCN-128, suggests the presence of absorption competition for the excitation light between the PCN-128 and OTC, and the inner filter effect (IFE) process is the reason for the fluorescence quenching here. Since the luminescence of PCN-128 originates from the H_4_ETTC ligand, the photoinduced electron transfer (PET) process between the H_4_ETTC ligand and OTC may quench the fluorescence of PCN-128^[1, 7]^. Specifically, electrons in the highest occupied molecular orbital (HOMO) of H_4_ETTC are excited to its lowest unoccupied molecular orbital (LUMO) and then transferred to the LUMO of OTC. To verify this, we calculated the relative energy levels of the HOMO and LUMO of H_4_ETTC and OTC. The LUMO energy level of H_4_ETTC (-2.90 eV) is higher than that of OTC (-3.00 eV), supporting the PET process from H_4_ETTC to OTC. Therefore, both the IFE and PET process contributed to the fluorescence quenching of PCN-128.

**
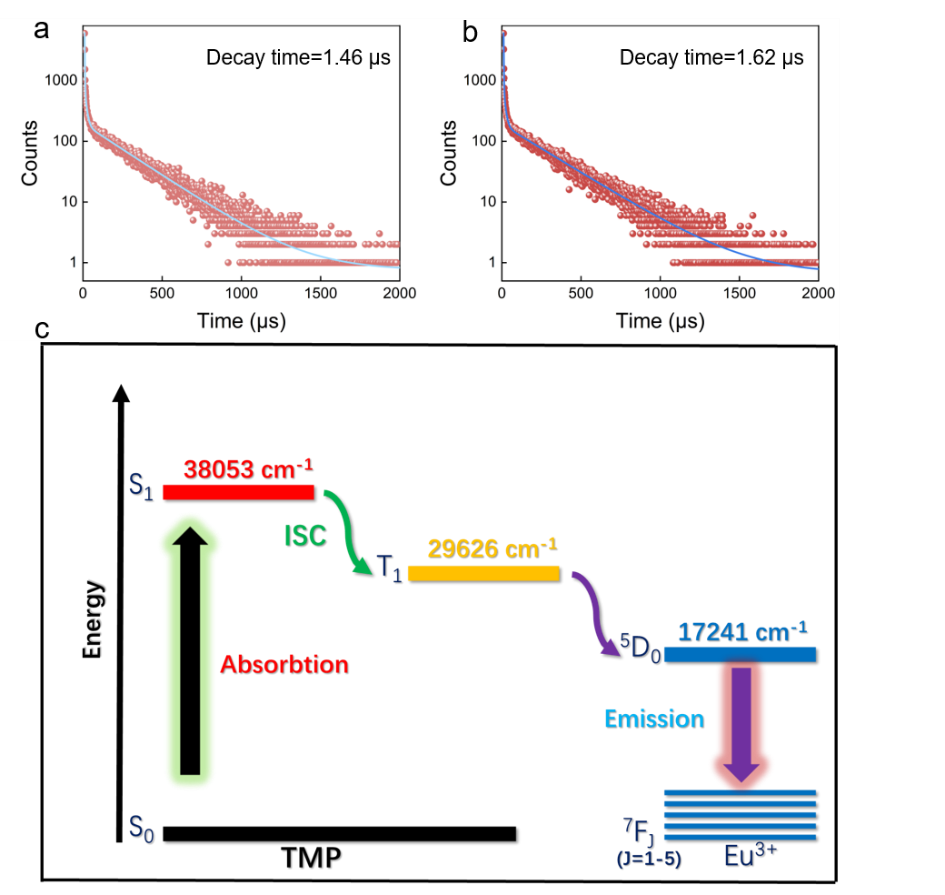
**

**Figure S12**. (a) Fluorescence lifetime curve for Eu-MOFs. (b) Fluorescence life fitting curve of Eu-MOFs soaked in TMP solution. (c) The energy transfer representation from TMP to Eu^3+^.

Compared to Eu-MOFs alone, the fluorescence lifetime after the reaction increased from 1.46 μs to 1.62 μs, which confirms that Eu-MOFs exhibit a fluorescence enhancement effect upon addition of TMP. Moreover, the strong peak of Eu^3+^ and the absence of emission from TMP indicate that the excitation energy of TMP can effectively transfer to the Eu^3+^ center, which can be attributed to the efficient antenna effect. According to Reinhoudt empirical rules, when the energy gap between the excited singlet and the excited triplet (Δ*E*_ST_) should be greater than 5,000 cm^−1^, and the energy gap between the ligand and the excited triplet and lanthanide ions (Δ*E*_T0_) should usually be higher than 3,500 cm^−1^ ^[8]^. Based on DFT calculations, the singlet state energy of TMP is 38053 cm^−1^, and the triplet state energy of TMP is 29626 cm^−1^, resulting in a Δ*E*_ST_ of 8427 cm^−1^. The excitation energy of Eu^3+^ is 17,241 cm^−1^, corresponding to a Δ*E*_T0_ of 12385 cm^−1^. Therefore, the excitation energy can effectively transfer from TMP to the Eu^3+^ center, which is attributed to the efficient antenna effect.


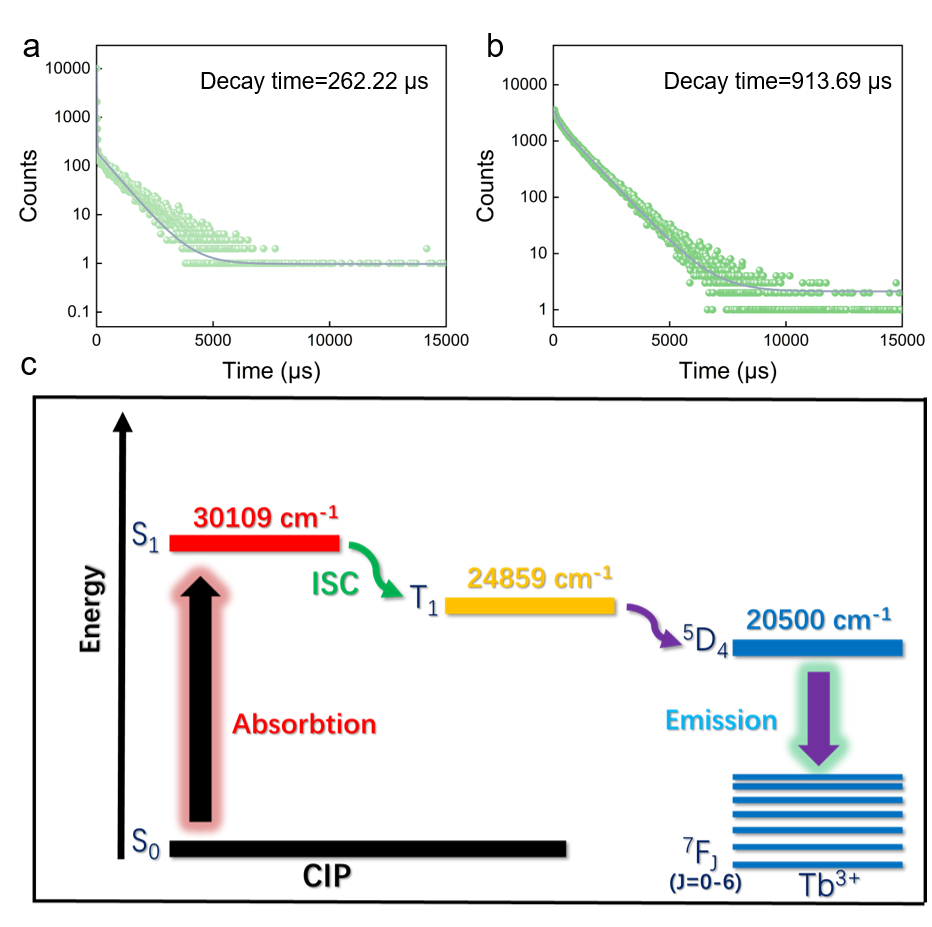


**Figure S13**. (a) Fluorescence lifetime curve for Tb-MOFs. (b) Fluorescence life fitting curve of Tb-MOFs soaked in CIP solution. (c) The energy transfer representation from CIP to Tb^3+^.

According to literature research, CIP can act as a bridging molecule, replacing the coordinating water molecules around Tb^3+^ of Tb-MOFs, and serve as an antenna molecule to transfer energy to Tb^3+^ ^[3, 9]^. At first, we measured the fluorescence lifetime of Tb-MOFs before and after reacting with CIP under 365 nm excitation. Compared to Tb-MOFs, the fluorescence lifetime after the reaction to CIP increased from 262.22 μs to 913.69 μs, which confirmed that Tb-MOFs exhibit a fluorescence enhancement effect upon addition of CIP. To verify the antenna effect of CIP, we employed DFT calculations to determine the energies of the excited singlet and triplet states of CIP, as well as the excited state energy of Tb^3+^. The singlet state energy of CIP is 30109 cm^-1^, while the triplet state energy is 24859 cm^-1^, resulting in a Δ*E*_ST_ of 5250 cm^-1^, which is higher than 5000 cm^-1^. The excitation energy of Tb^3+^ is 20500 cm^-1^, and the Δ*E*_T0_ with the triplet state of CIP is 4359 cm^-1^, which is also greater than 3,500 cm^-1^. This demonstrated the strong antenna effect of CIP, leading to the fluorescence enhancement of Tb-MOFs.

**
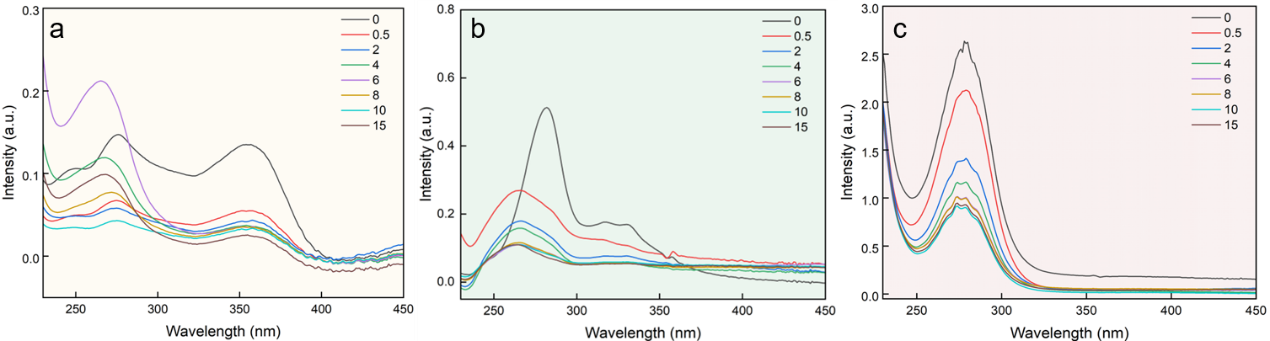
**

**Figure S14.** Adsorption curves of (a) OTC, (b) TMP, (c) CIP based on different MOFs at different times (0-15 min).

**
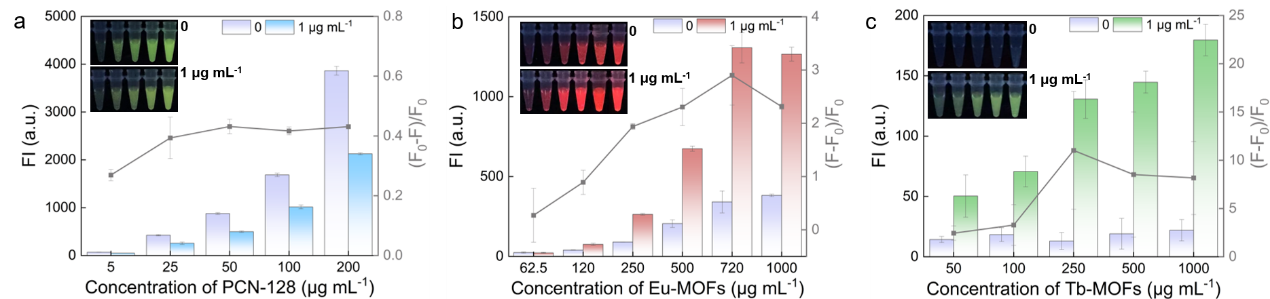
**

**Figure S15.** Fluorescence intensity and QRs (ERs) of different concentration of (a) PCN-128, (b) Eu-MOFs and (c) Tb-MOFs to OTC, TMP, and CIP for 10 min, respectively.

The quantity of the MOFs for fluorescence detection is one of the most important parameters that determines the performance. Hence, we optimized the amount of three kinds of MOFs via controlling the concentration by comparing quenching rates (QRs) or enhancement rates (ERs) of different MOFs towards antibiotics. The rise of QRs or ERs values can be controlled by adjusting the amount of different MOFs, and 50 μg mL^-1^, 720 μg mL^-1^ and 250 μg mL^-1^ were chosen as the optimized parameter for PCN-128, Eu-MOFs and Tb-MOFs, respectively.

**
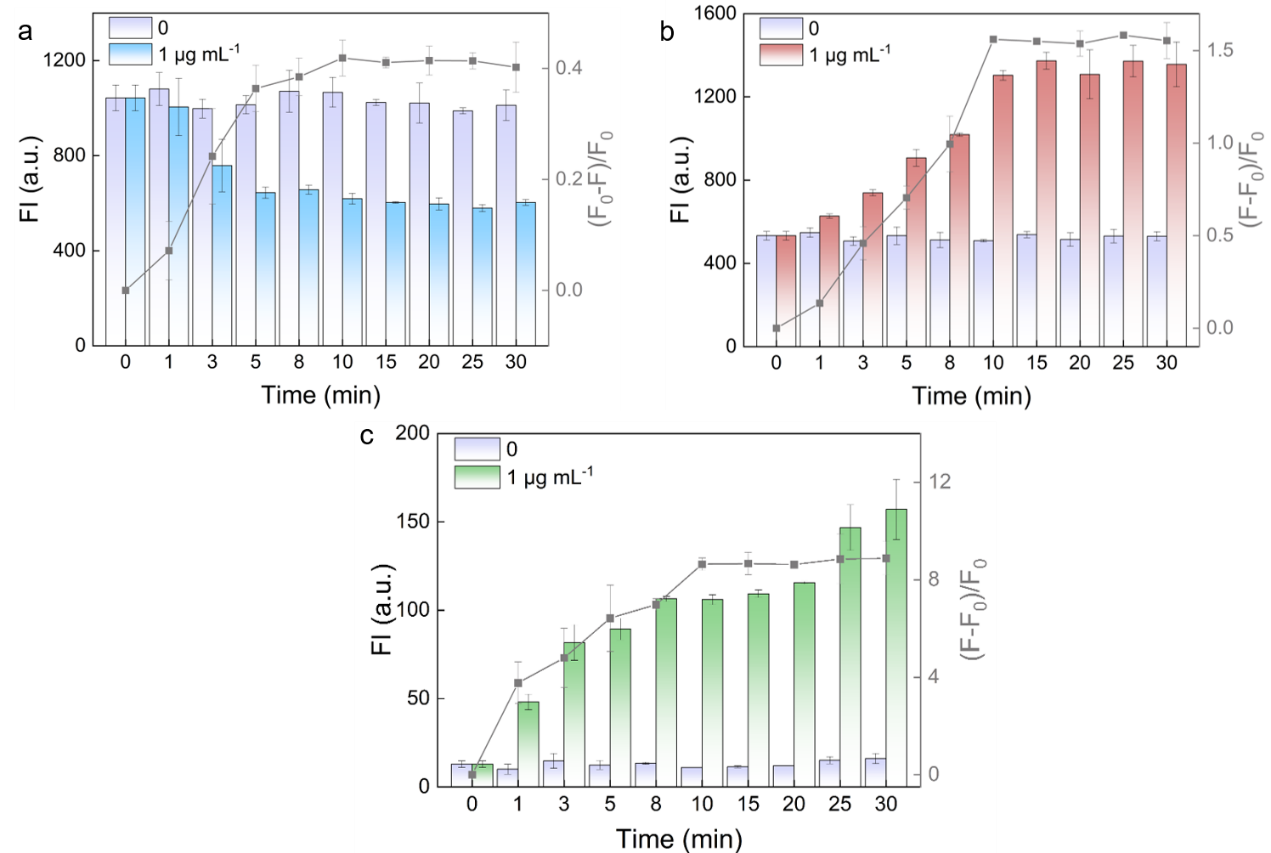
**

**Figure S16.** Fluorescence intensity and QRs (ERs) of (a) PCN-128, (b) Eu-MOFs and (c) Tb-MOFs to OTC, TMP, and CIP for different time, respectively.

With the extension of detection time of antibiotics by different MOFs, both QRs or ERs values increased and stabilized at 10 min. Therefore, 10 min was chosen as the optimal detection time.

**
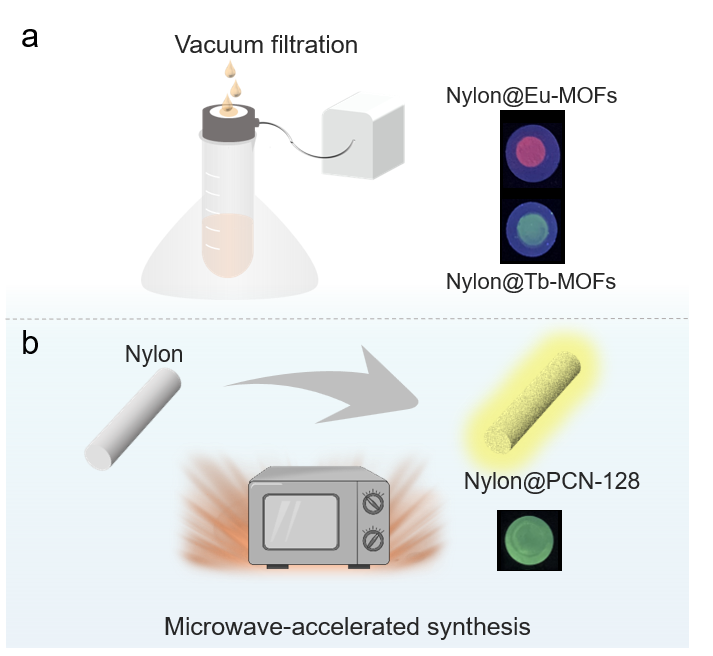
**

**Figure S17.** Schematic of preparation process of Nylon@Eu-MOFs, Nylon@Tb-MOFs and Nylon@PCN-128. (a) Vacuum-assisted filtration, (b) Microwave-accelerated synthesis.

Nylon@Eu-MOFs and Nylon@Tb-MOFs were prepared by the simple vacuum-assisted filtration method based on the synthesized MOFs powders. Nylon@PCN-128 was prepared by microwave assisted solvothermal method directly in the precursor solution.

**
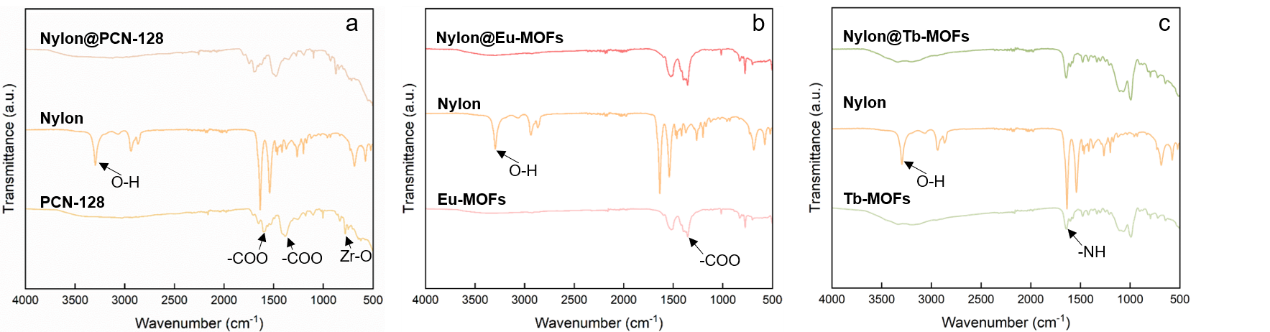
**

**Figure S18.** FT-IR characterizations of different MOFs and the corresponding hybrid membranes. (a) PCN-128, (b) Eu-MOFs and (c) Tb-MOFs.

**
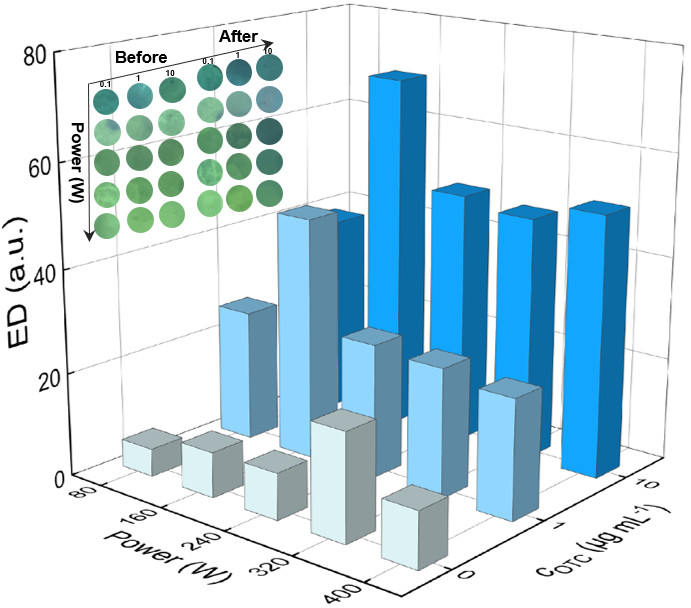
**

**Figure S19.** The changes of ED values of Nylon@PCN-128 created by different microwave power to 1 μg mL^-1^ OTC in ACN-H_2_O.

The intensity of fluorescence images of the Nylon@PCN-128 surface was uniform, with better detection performance at the power of 160 W.

**
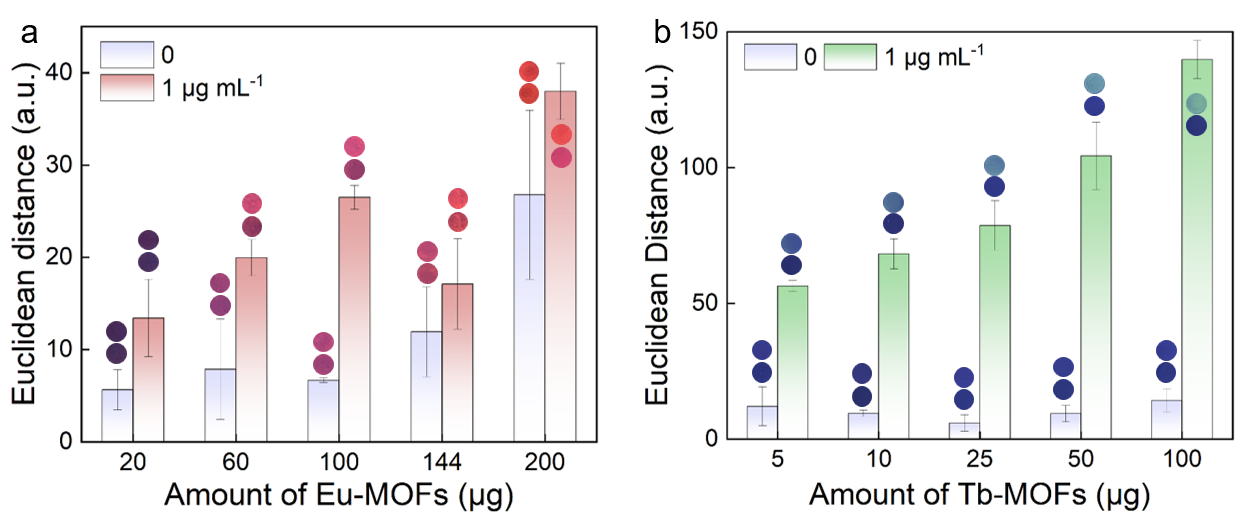
**

**Figure S20.** The changes of ED values of different MOFs hybrid membranes with different amount of MOFs to 1 μg mL^-1^ target antibiotics, (a) Eu-MOFs, (b) Tb-MOFs.

We optimized the loading amount of Eu-MOFs and Tb-MOFs for the Nylon@Eu-MOFs and Nylon@Tb-MOFs to achieve the excellent response. The EDs initially increased and then decreased within the range from 20 to 200 μg, leading to the selection of 100 µg as the optimal volume for Nylon@Eu-MOFs. The Nylon membrane with a higher loading amount of Tb-MOFs exhibited a stronger response to CIP. Based on the principle of sustainability and resource efficiency, 50 µg was chosen as the optimal filtration volume parameter for Nylon@Tb-MOFs.

**
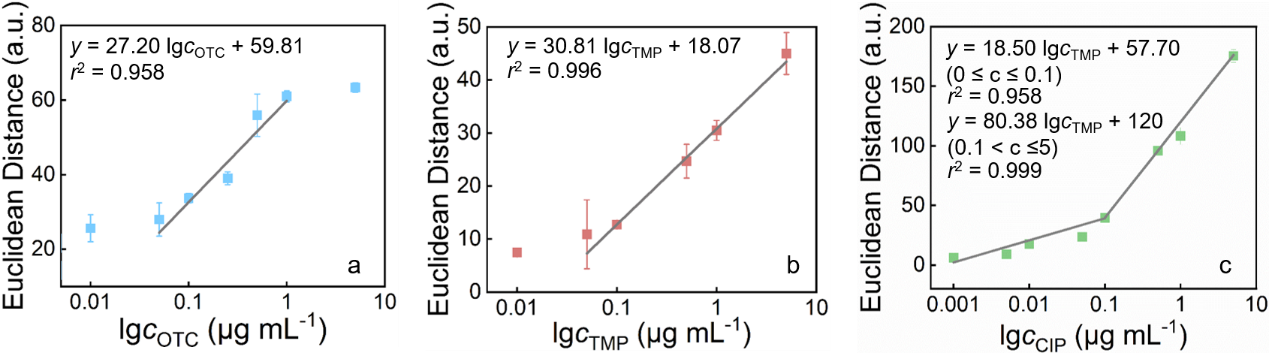
**

**Figure S21.** The calibration plot of different MOFs hybrid membrane towards different concentrations of target antibiotics in ACN-H_2_O. (a) Nylon@PCN-128, (b) Nylon@Eu-MOFs and (c) Nylon@Tb-MOFs.

The Nylon@PCN-128 sensor presented a LDR from 0.05 to 1 μg mL^-1^ and a LOD of 0.0085 μg mL^-1^ (*S*/*N*=3, **a**), which is about seven times lower than that based on PCN-128 powders and further indicated the outstanding detection performance of hybrid membrane. Similarly, the LDR of Nylon@Eu-MOFs sensor is 0.05 - 5 μg mL^-1^, with the lower LOD (0.0225 μg mL^-1^, *S*/*N*=3) than Eu-MOFs (**b**). Moreover, the ED values showed a strong correlation with the concentration of CIP in the ranges of 0 -0.1 μg mL^-1^ and 0.1-5 μg mL^-1^ (**c**), with a LOD of 0.00136 μg mL^-1^ (*S*/*N*=3), which is lower than those of other MOFs-based fluorescent sensors ^[3, 10-13]^.

**
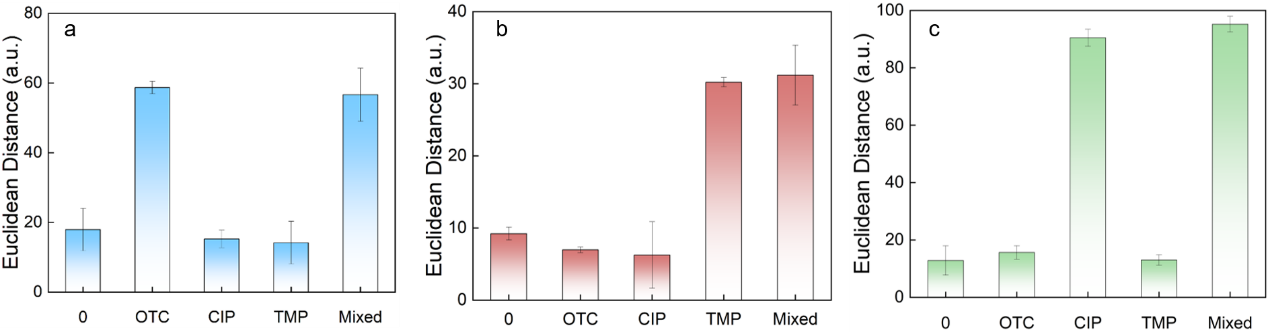
**

**Figure S22.** ED values of (a) Nylon@PCN-128, (b)Nylon@Eu-MOFs, and (c) Nylon@Tb-MOFs to different antibiotics at 1 μg mL^-1^.

**
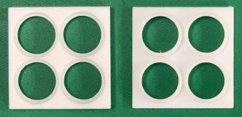
**

**Figure S23.** The custom-designed support component of MOFs membrane sensor array created by computerized numerical control.


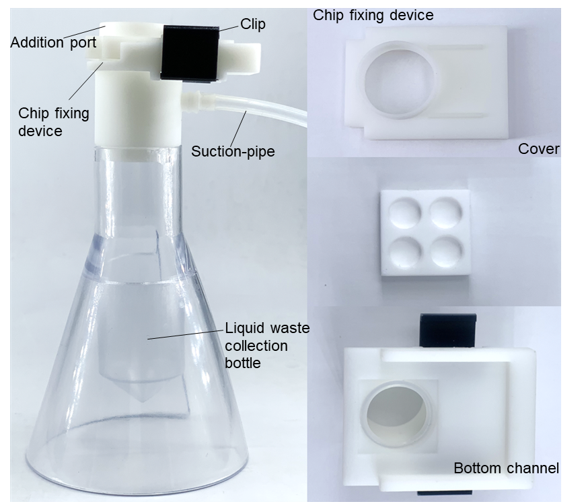


**Figure S24.** Portable vacuum filtration device compatible with the MOFs membrane sensor array for high-throughput detection.

The portable miniaturized filtration device consisted of an addition port, clamping fixing device, clip, suction-pipe, and liquid waste collection bottle, arranged from top to bottom. All components, except for the vacuum tube, were fabricated using 3D printing technology, which is both well-established and cost-effective.

**Table S1.** Summary of sensor arrays for simultaneous detection of multiple antibiotics.

| **Sensing elements** | **Targets** | **Detection** | **Real sample** | **Ref.** |
| --- | --- | --- | --- | --- |
| Fluorophores | Aminoglycoside  (amikacin, kanamycin,  streptomycin, gentamycin, tobramycin, neomycin)  (1.34 ppm) | Deep learning | / | ^[14]^ |
| Luminol/  fluorescein | Diethylstilbestro, metronidazole, kanamycin, isoniazide,  ceftiofur sodium | HCA/PCA  DNN | / | ^[15]^ |
| Carbon dots | Tetracyclines (tetracycline, OTC, aureomycin)  Aminoglycosides (streptomycin, neomycin)  Amide alcohols (chloramphenicol) Macrolides (spiramycin, erythromycin) | LDA | Human urine | ^[16]^ |
| Carbon dots | Nitroimidazole  (metronidazole, ornidazole, tinidazole, secnidazole, ronidazol, metronidazole) | LDA | Milk  Honey | ^[17]^ |
| Carbon nanoparticles | β-lactam (ampicillin)  Quinolones (CIP)  Aminoglycosides (kanamycin)  Sulfonamides (sulphamethoxazole)  Tetracyclines (tetracycline) | Deep learning | Poultry feeds | ^[18]^ |
| Carbon dots/  Quantum dots | Fluoroquinolones (ofloxacin, pefloxacin mesylate, norfloxacin) Aminoglycosides (amikacin, kanamycin, streptomycin)  Tetracyclines (doxycycline, tetracycline, metacycline) | Machine learning | River water  Milk | ^[19]^ |
| Eu^3+^/Al^3+^ | Tetracyclines  (OTC, tetracycline, doxycycline, chlortetracycline) | LDA | Tap/  lake water | ^[20]^ |
| Eu^3+^/Tb^3+^-MOFs | Minocycline, 1.23 μM;  norfloxacin, 0.06 μM | LDA/PCA | Drugs  Milk | ^[21]^ |
| Pyrene-based MOFs | Sulfonamide  (sulfamethoxazole, sulfadiazine, sulfapyridine, sulfamerazine, sulfamethazine)  (10^-8^-10^-7^ moL L^−1^) | LDA | Real water | ^[22]^ |
| RhB@PCN-222 | Tetracyclines  (0.15-0.17 μM) | LDA | Milk | ^[23]^ |

HCA=hierarchical clustering analysis; PCA= principal component analysis;

DNN=deep neural networks; LDA=linear discriminant analysis.

**Reference**

[1] Y. Zhou, Q. Yang, D. Zhang, N. Gan, Q. Li, J. Cuan, *Sens. Actuators B: Chem.* **2018**, *262*, 137.

[2] J. Yan, J. Zhang, M. Zhang, G. Shi, *Talanta* **2022**, *237*, 122920.

[3] H. Wang, X. Qian, X. An, *Carbohydr. Polym.* **2022**, *287*, 119337.

[4] Q. Q. Li, M. J. Wen, Y. S. Zhang, Z. S. Guo, X. Bai, J. X. Song, P. Liu, Y. Y. Wang, J. L. Li, *J. Hazard. Mater.* **2022**, *423*, 127132.

[5] X. Sun, Y. Qiao, M. Zhang, Y. Cheng, F. Ning, H. Zhang, P. Hu, *Microchem. J.* **2023**, *190*, 108687.

[6] X. Kang, Z. Jiao, X. Shi, Y. Tian, Z. Liu, *J. Mater. Chem. C* **2022**, *10* (42), 16078.

[7] L. Liu, Q. Chen, J. Lv, Y. Li, K. Wang, J.-R. Li, *Inorg. Chem.* **2022**, *61* (20), 8015.

[8] S. Su, D. Li, Y. Yang, R. Xu, W. Hu, W. Li, M. Hu, *Appl. Organomet. Chem.* **2024**, *38* (7), e7524.

[9] B. Liu, Y. Huang, X. Zhu, Y. Hao, Y. Ding, W. Wei, Q. Wang, P. Qu, M. Xu, *Anal. Chim. Acta* **2016**, *912*, 139.

[10] X. Yue, C. Wu, Z. Zhou, L. Fu, Y. Bai, *Foods* **2022**, *11* (19), 3138.

[11] C.-Y. Wang, C.-C. Wang, X.-W. Zhang, X.-Y. Ren, B. Yu, P. Wang, Z.-X. Zhao, H. Fu, *Chin. Chem. Lett.* **2022**, *33* (3), 1353.

[12] B. Wang, B. Yan, *Talanta* **2020**, *208*, 120438.

[13] K. Zhu, R. Fan, X. Zheng, P. Wang, W. Chen, T. Sun, S. Gai, X. Zhou, Y. Yang, *J. Mater. Chem. C* **2019**, *7* (47), 15057.

[14] X. Tan, Y. Liang, Y. Ye, Z. Liu, J. Meng, F. Li, *Anal. Chem.* **2022**, *94* (2), 829.

[15] F. Li, M. Zhu, Z. Li, N. Shen, H. Peng, B. Li, J. He, *Talanta* **2024**, *269*, 125446.

[16] Y. Mao, S. Cui, W. Li, X. Fan, Y. Liu, S. Xu, X. Luo, *Sens. Actuators B: Chem.* **2019**, *296*, 126694.

[17] S. Wang, Y. Wang, Y. Ning, W. Wang, Q. Liu, *Talanta* **2024**, *271*, 125679.

[18] S. Mandal, D. Paul, S. Saha, P. Das, *Sens. Actuators B: Chem.* **2022**, *360*, 131660.

[19] Z. Xu, K. Wang, M. Zhang, T. Wang, X. Du, Z. Gao, S. Hu, X. Ren, H. Feng, *Sens. Actuators B: Chem.* **2022**, *359*, 131590.

[20] Y. Zhang, T. Wang, H. Guo, X. Gao, Y. Yan, X. Zhou, M. Zhao, H. Qin, Y. Liu, *Biosens. Bioelectron.* **2023**, *231*, 115266.

[21] R. Xie, P. Yang, J. Liu, X. Zou, Y. Tan, X. Wang, J. Tao, P. Zhao, *Talanta* **2021**, *231*, 122366.

[22] Z. Yang, J. Tang, B. Chen, X. Qu, H. Fu, *ACS Appl. Nano Mater.* **2023**, *6* (24), 23245.

[23] W.-T. Li, J.-S. Wang, M. Pang, Y. Li, W.-J. Ruan, *Sens. Actuators B: Chem.* **2023**, *381*, 133375.
